# Dynamic Metalloporphyrin‐Based [2]Rotaxane Molecular Shuttles Stimulated by Neutral Lewis Base and Anion Coordination

**DOI:** 10.1002/chem.202300608

**Published:** 2023-04-26

**Authors:** Jamie T. Wilmore, Yuen Cheong Tse, Andrew Docker, Caspar Whitehead, Charlotte K. Williams, Paul D. Beer

**Affiliations:** ^1^ Department of Chemistry University of Oxford Chemistry Research Laboratory Mansfield Road Oxford OX1 3TA UK

**Keywords:** dynamic rotaxanes, metalloporphyrins, molecular shuttles, rotaxanes, supramolecular

## Abstract

A series of dynamic metalloporphyrin [2]rotaxane molecular shuttles comprising of bis‐functionalised Zn(II) porphyrin axle and pyridyl functionalised macrocycle components are prepared in high yield via active metal template synthetic methodology. Extensive variable temperature ^1^H NMR and quantitative UV‐Vis spectroscopic titration studies demonstrate dynamic macrocycle translocation is governed by an inter‐component co‐ordination interaction between the macrocycle pyridyl and axle Zn(II) metalloporphyrin, which serves to bias a ‘resting state’ co‐conformation. The dynamic shuttling behaviour of the interlocked structures is dramatically inhibited by the addition of a neutral Lewis base such as pyridine, but can also be tuned via post‐synthetic rotaxane demetallation of the porphyrin axle core to give free‐base, or upon subsequent metallation, Ni(II) [2]rotaxane analogues. Importantly, the Lewis acidic Zn(II) porphyrin axle component is also capable of coordinating anions which induces mechanical bond shuttling behaviour resulting in a novel optical sensing response.

## Graphical abstract

**A series of dynamic** Zn(II), Ni(II) and free base porphyrin‐containing rotaxanes are reported. Co‐ordination to the Zn(II) axle porphyrin centre by a macrocycle‐contained pyridyl Lewis base affords significant co‐conformational bias. Displacement of the macrocycle by a competing neutral Lewis base or anion elucidates an optical response in the porphyrin absorption spectra, enabling a novel optical signal transduction mechanism for dynamic mechanical bond shuttling and anion detection.

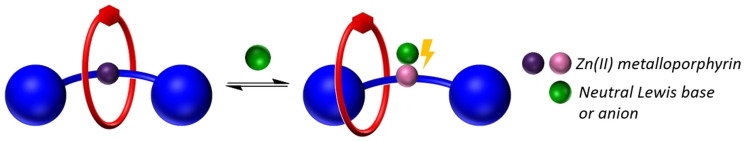

## Introduction

Recent years have witnessed the exploitation of mechanically interlocked molecules (MIMs) in a diverse range of applications including molecular recognition,[^1^, ^2^, ^3^, ^4^, ^5^, ^6^] sensing[^7^, ^8^, ^9^, ^10^] and catalysis,[^11^, ^12^, ^13^, ^14^] wherein the unique microenvironment or topology of the mechanical bond underpins the functional behaviour of the system.[^15^, ^16^, ^17^, ^18^] The exquisite topological control afforded by the mechanical bond in MIMs frequently affords enhanced host‐guest binding behaviour, with increased selectivity and binding affinities over their non‐interlocked analogues.^[19]^

The inherent dynamic motion of MIMs resulting from the interlocked, yet not covalently joined, nature of macrocycle and axle components has been exploited to achieve stimuli responsive switchable control over the location of the macrocycle on the axle, resulting in significant bias in the location of the macrocycle between one or more shuttling stations, affording distinct co‐conformations in MIMs.[^20^, ^21^, ^22^] The introduction of electrochemical and photochemical reporter groups, whose response is dependent upon the inter‐component separation, allows measurement of relative co‐conformation occupancy, and by extension, the presence or absence of the motion‐inducing stimulus.[^23^, ^24^]

The prevalence of anions in biological and environmental processes has led to marked interest in the sensing of anions. We and others have exploited controllable dynamic MIM behaviour via an anion recognition stimulus.[^25^, ^26^, ^27^, ^28^, ^29^, ^30^, ^31^, ^32^, ^33^] Typically, such dynamic motion arises from an anion being bound in the cavity between macrocycle and axle components. Systems in which the guest directly displaces a macrocycle from a ‘resting state’ co‐conformation with significant macrocycle‐axle binding affinity remain rare.[^34^, ^35^]

Metalloporphyrins have proven a valuable supramolecular synthon as a modular Lewis acidic centre capable of binding of neutral and anionic guests.[^36^, ^37^, ^38^, ^39^, ^40^] Advantageously, these host‐guest recognition events are often relayed through perturbations in the metalloporphyrin's photophysical or electrochemical properties which facilitates signal transducing responsive sensing capability.[^41^, ^42^, ^43^, ^44^, ^45^] This has recently been exploited with the integration of metalloporphyrins into MIM rotaxane structural host design to produce systems capable of exhibiting optical or electrochemical responsive switchable co‐conformational shuttling behaviour.[^46^, ^47^]

**Figure 1:**
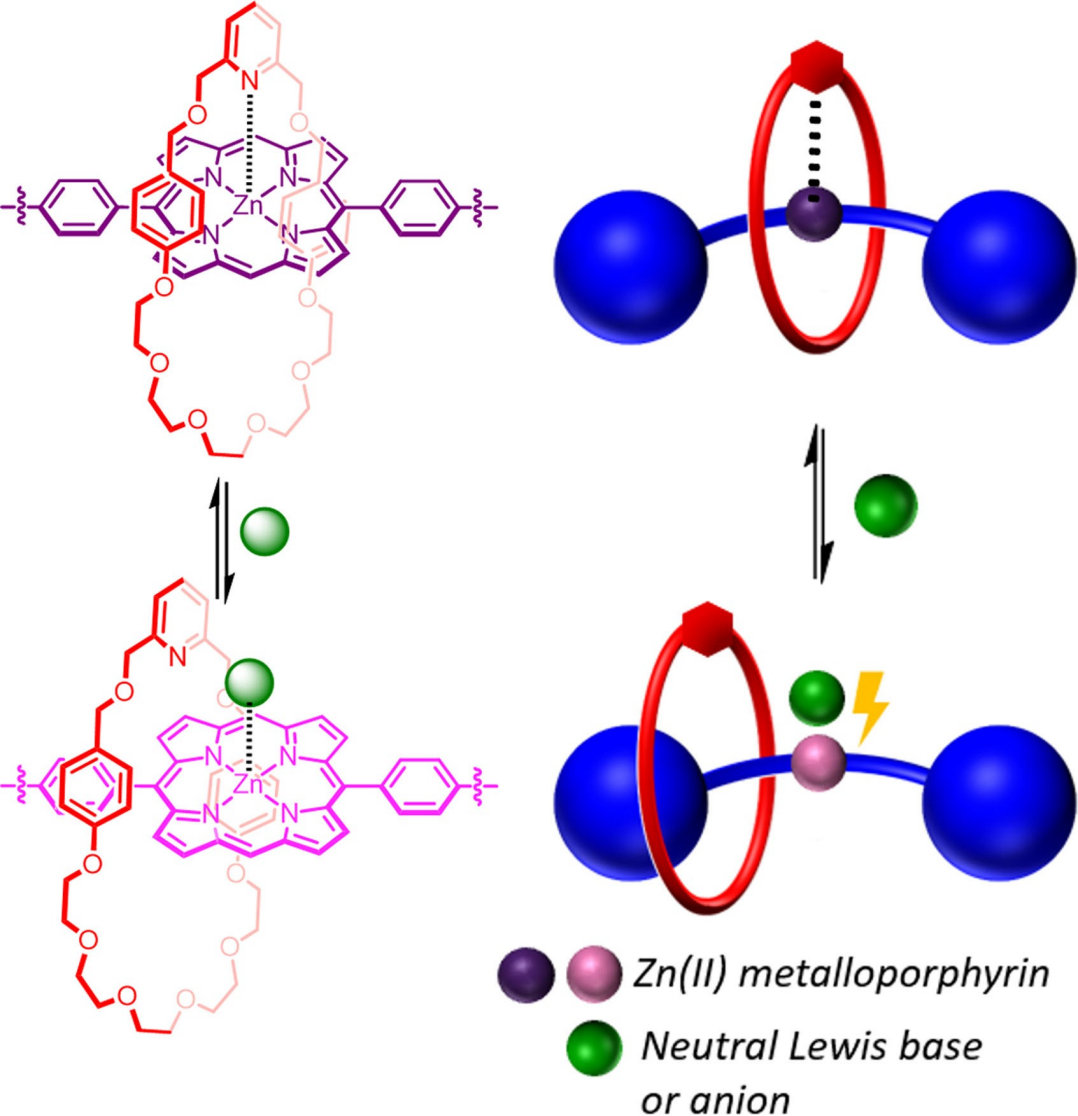
Cartoon representation of competitive neutral Lewis base or anion co‐ordination inducing dynamic shuttling behaviour signalled via an optical response in the metalloporphyrin‐containing [2]rotaxane.

Herein, we report the high yielding synthesis and characterisation of a series of dynamic metalloporphyrin based [2]rotaxanes, comprising of a pyridyl functionalised macrocycle and an axle with a central zinc(II) metalloporphyrin. NMR and UV‐visible spectroscopic investigations reveal such an interlocked structure possesses an inter‐component macrocycle pyridyl⋅⋅⋅Zn(II) porphyrin axle co‐ordination interaction, imparting significant bias towards a ‘resting state’ co‐conformation in which the macrocycle co‐ordinates to the axle. The disruption of this binding interaction through the competitive binding of either a neutral pyridine ligand or co‐ordinating halide anion guest species, leads to displacement, and therefore translocation, of the macrocycle. Importantly, the metalloporphyrin‐anion binding event results in an optical response in the rotaxane axle's porphyrin‐based absorption which serves as a novel transduction mechanism for signalling mechanical bond dynamic shuttling behaviour and detecting anions (Figure 1).

## Results and Discussion

### Rotaxane synthesis

**Scheme 1:**
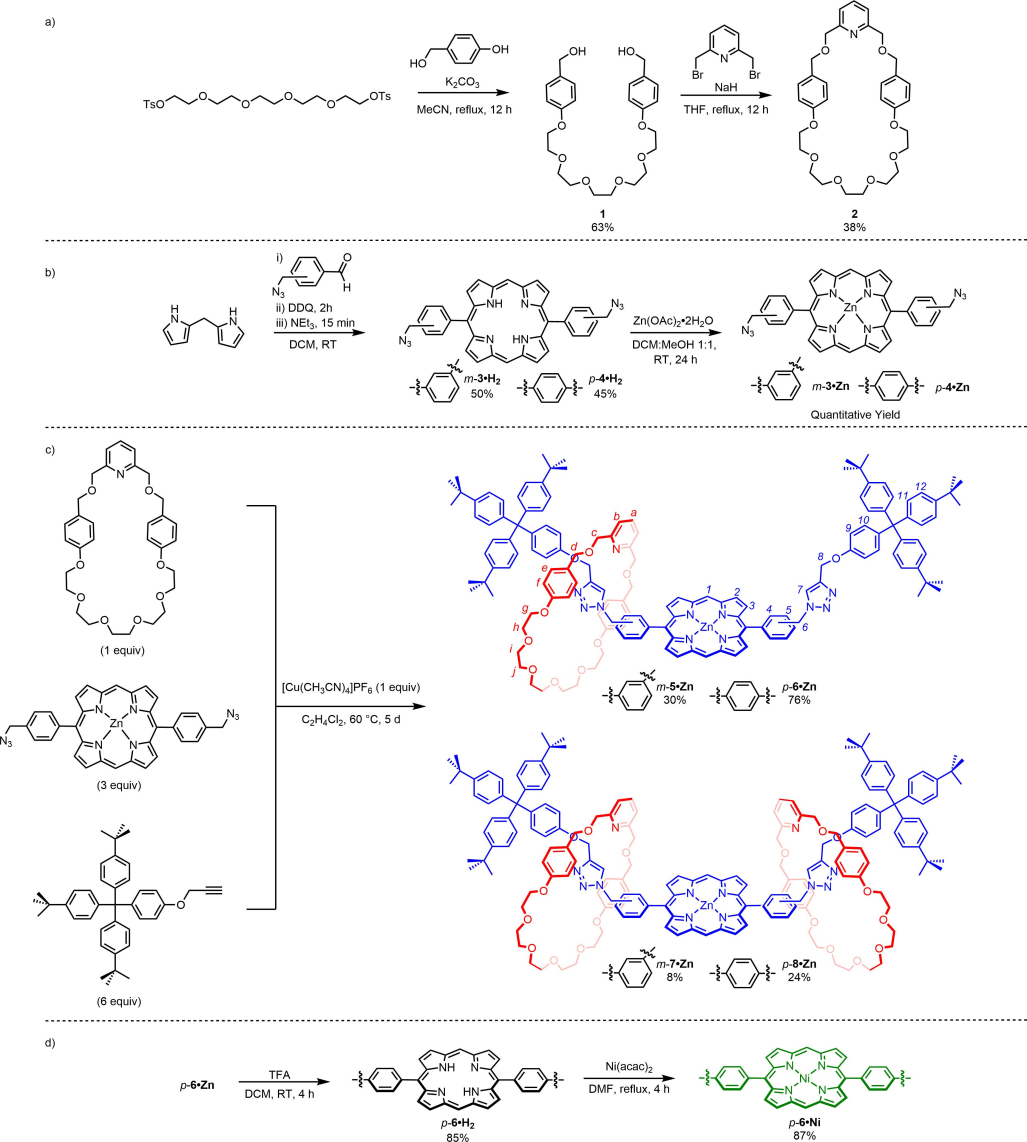
Synthesis of a) macrocycle **2**, b) Zn(II) metalloporphyrin bis‐azides *m*‐**3 ⋅ Zn** and *p*‐**4 ⋅ Zn**, c) [2]rotaxanes *m*‐**5 ⋅ Zn** and *p*‐**6 ⋅ Zn**, and [3]rotaxanes *m*‐**7 ⋅ Zn** and *p*‐**8 ⋅ Zn**, and d) Ni(II) metalloporphyrin containing rotaxane *p*‐**6 ⋅ Ni**.

The target [2]rotaxane structures were prepared via a Cu(I) azide alkyne cycloaddition (CuAAC) active metal template (AMT) methodology,[^48^, ^49^, ^50^, ^51^] employing a pyridyl functionalised macrocycle and regioisomeric bis‐azide phenyl appended Zn(II) porphyrin axle precursors. The requisite macrocycle was synthesised according to Scheme 1a. Specifically, 4‐hydroxybenzyl alcohol was reacted with pentaethylene glycol ditosylate, in acetonitrile solution with K_2_CO_3_, to afford diol **1** in 63 % yield, after purification by column chromatography. A subsequent pseudo high‐dilution Williamson ether macrocyclisation reaction between diol **1** and 2,6‐bis‐bromomethylpyridine, in the presence of NaH, gave the desired pyridyl macrocycle **2** in 38 % yield.

The requisite bis‐azide appended Zn(II) porphyrin precursors were synthesised according to Scheme 1b. For both *meta*‐ and *para*‐bis‐azide phenyl substituted porphyrin precursors, the free‐base porphyrin was synthesised via an acid‐catalysed condensation reaction between the appropriately functionalised azide appended benzaldehyde and dipyrrole methane, followed by a DDQ mediated oxidation. Purification by column chromatography of the crude products afforded the regioisomeric *meta‐* and *para‐* bis‐azide phenyl substituted free base porphyrins *m‐* **3 ⋅ H_2_** and *p‐* **4 ⋅ H_2_** in yields of 50 % and 45 %, respectively.^[52]^ Subsequent zinc complexation was achieved through stirring the free‐base porphyrins with Zn(OAc)_2_ ⋅ 2H_2_O, in 1 : 1 *v/v* CH_2_Cl_2_ : MeOH, to afford the target Zn(II) metalloporphyrin bis‐azide axle precursors *m‐* **3 ⋅ Zn** and *p‐* **4 ⋅ Zn** in quantitative yield.

With the required components in hand, a general AMT MIM CuAAC synthetic method[^48^, ^49^, ^50^, ^51^, ^52^] was employed for rotaxane construction, in which either *m‐* **3 ⋅ Zn** or *p‐* **4 ⋅ Zn** and terphenyl functionalised stopper alkyne component^[53]^ were added to a solution of macrocycle precomplexed with a catalytically active Cu(I) source, Cu(MeCN)_4_PF_6_, in 1,2‐dichloroethane (DCE) and the reaction mixture left to stir at 60 °C (Scheme 1c).

In both cases, after 5 days, analysis of the crude reaction mixture by TLC indicated complete consumption of the bis‐azide starting materials and ESI‐MS analysis of the crude reaction mixtures revealed signals at *m/z*=2274 corresponding to the target isomeric [2]rotaxanes. Subsequent purification by preparative thin layer chromatography gave the desired target *meta‐* and *para‐* [2]rotaxanes, *m*‐**5 ⋅ Zn** and *p‐* **6 ⋅ Zn**, in yields of 30 % and 76 %, respectively, in addition to the corresponding non‐interlocked axle components (*m*‐**9 ⋅ Zn**, *p*‐**10 ⋅ Zn**). The isolated [2]rotaxanes and non‐interlocked axles were characterised by ^1^H,^13^C{^1^H} NMR spectroscopy and high‐resolution electrospray ionisation (HR‐ESI) mass spectrometry (See Supporting Information).

**Figure 2:**
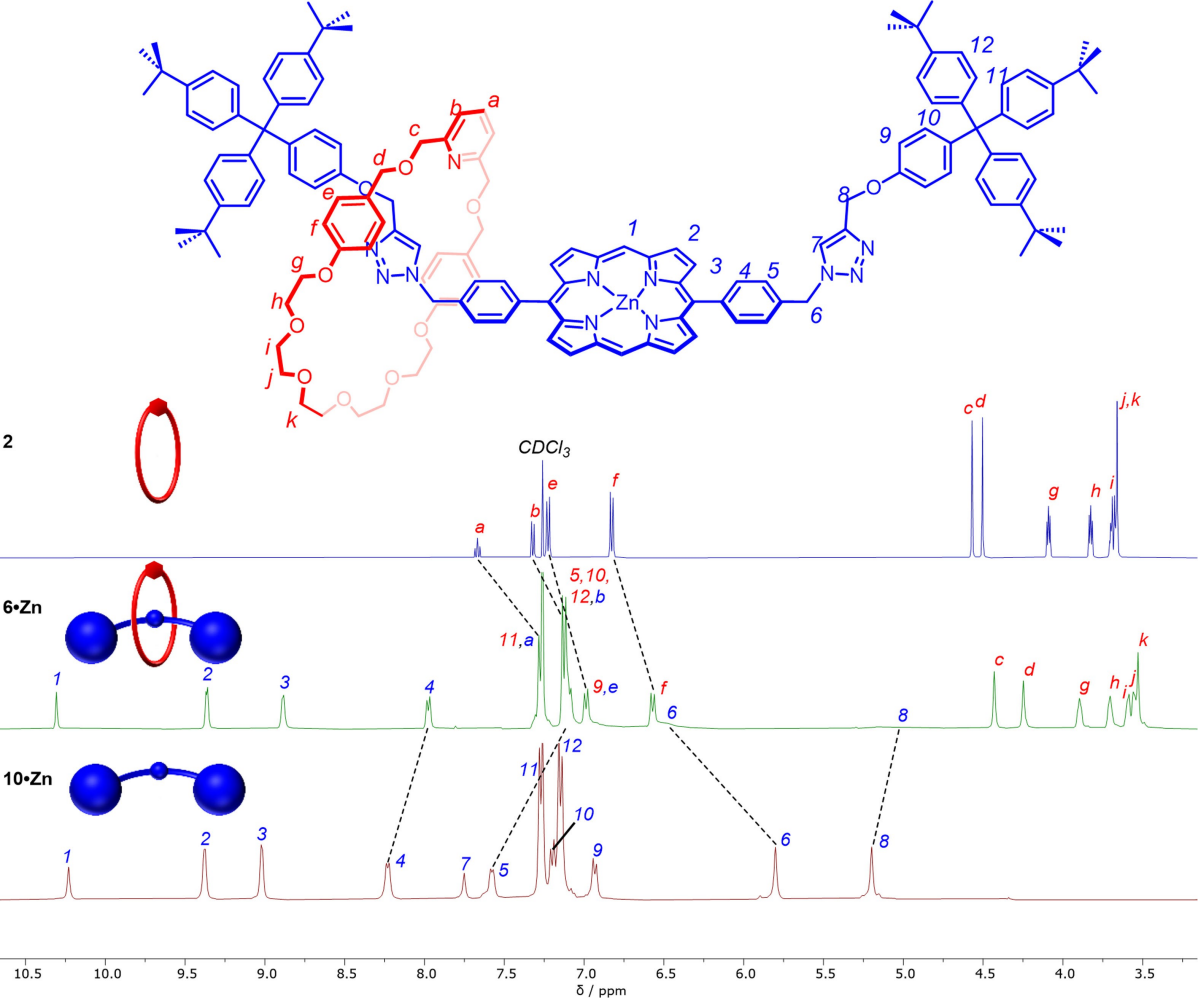
Stacked ^1^H NMR Spectra (400 MHz, CDCl_3_, 298 K) of macrocycle **2**, [2]rotaxane *p*‐**6 ⋅ Zn** and non‐interlocked axle *p*‐**10 ⋅ Zn**.

The interlocked nature of both [2]rotaxanes was confirmed through comparison of their ^1^H NMR spectra in CDCl_3_ with the constituent non‐interlocked components, a representative example of which for *p*‐**6 ⋅ Zn** is shown in Figure 2. Notably, the axle phenyl protons *H*_4_ and *H*_5_ display a downfield shift compared to the free axle, likely due to ring current effects from proximal macrocycle aromatic protons and a corresponding significant upfield shift (Δδ≈0.25 ppm) was observed in the macrocycle phenyl protons. Perhaps most notable is the significant broadening of the axle methylene signals *H*_6_ and *H*_8,_ and the complete disappearance of the triazole signal *H*_7_.

Interestingly, another species of higher polarity than the desired [2]rotaxanes was also consistently observed during preparative TLC purification. Evidently containing porphyrin, these additional products isolated from the CuAAC‐AMT reaction mixtures gave highly symmetrical and well resolved ^1^H NMR spectra displaying proton signals from a porphyrin axle and a macrocycle component, which upon integration revealed a 1 : 2 ratio respectively. ESI‐MS analysis confirmed the products were the higher order [3]rotaxanes where the porphyrin containing axles are encircled by two macrocycles. It is noteworthy that the isolated yields of the [3]rotaxanes was considerable (8 % and 24 % for *m*‐**7 ⋅ Zn** and *p*‐**8 ⋅ Zn** respectively) which translates to an impressive near quantitative total interlocked product yield of >99 % in the *para* case (Scheme 1c).

### Investigation of rotaxane inter‐component interactions

The observed broadness of the ^1^H NMR spectra for [2]rotaxanes *m‐* **5 ⋅ Zn** and *p‐* **6 ⋅ Zn** was attributed to a significant barrier to macrocycle translocation across the length of the axle. It was postulated that the internal pyridine moiety of the macrocycle was co‐ordinating to the zinc (II) Lewis acidic centre of the centrally situated metalloporphyrin‐containing axle.^[54]^ Indeed, such a MIM inter‐component interaction was noted by Mullen and co‐workers^[55]^ in their report of Zn(II) porphyrin‐axle stoppered rotaxanes containing a pyridyl functionalised macrocycle and in analogous gel resin functionalised [2]rotaxane materials.

**Figure 3:**
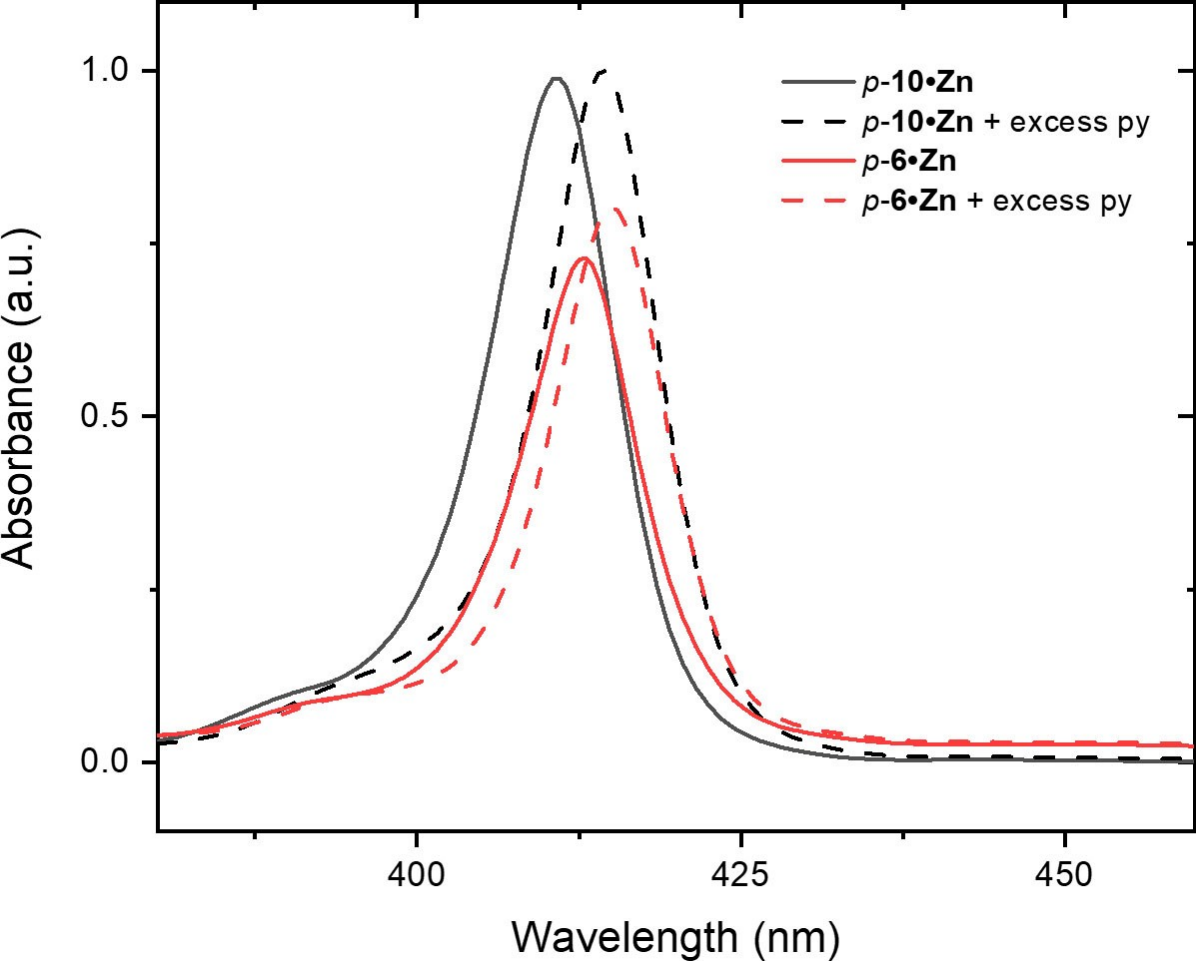
Soret‐band absorption spectra of *p*‐**6 ⋅ Zn** and the analogous non‐interlocked axle *p‐* **10 ⋅ Zn** in CH_2_Cl_2_ in the presence and absence of excess pyridine.

To gain further evidence for this co‐ordination‐induced conformational bias, preliminary UV‐Vis measurements of the MIMs and their non‐interlocked axle components were recorded in CH_2_Cl_2_ solution. Notably for both regioisomeric [2]rotaxanes *m*‐**5 ⋅ Zn** and *p*‐**6 ⋅ Zn** the Soret bands are bathochromically shifted in comparison to their corresponding axles. Such a shift is typically observed upon co‐ordination of the zinc(II) metalloporphyrin centre with a Lewis base (Figure 3, *p‐* **6 ⋅ Zn** Soret band λ_max_=413 nm, compared with the axle λ_max=_410 nm.)[^56^, ^57^] The addition of excess pyridine to a CH_2_Cl_2_ solution of either of the [2]rotaxanes or axles induced similar bathochromic Soret band shifts in λ_max_. However, the relative magnitude of shift in λ_max_ of the respective rotaxane is less than that of the corresponding axle, which is consistent with an already present inter‐component macrocycle‐pyridyl‐Zn(II) metalloporphyrin axle interaction in the interlocked systems.

**Figure 4:**
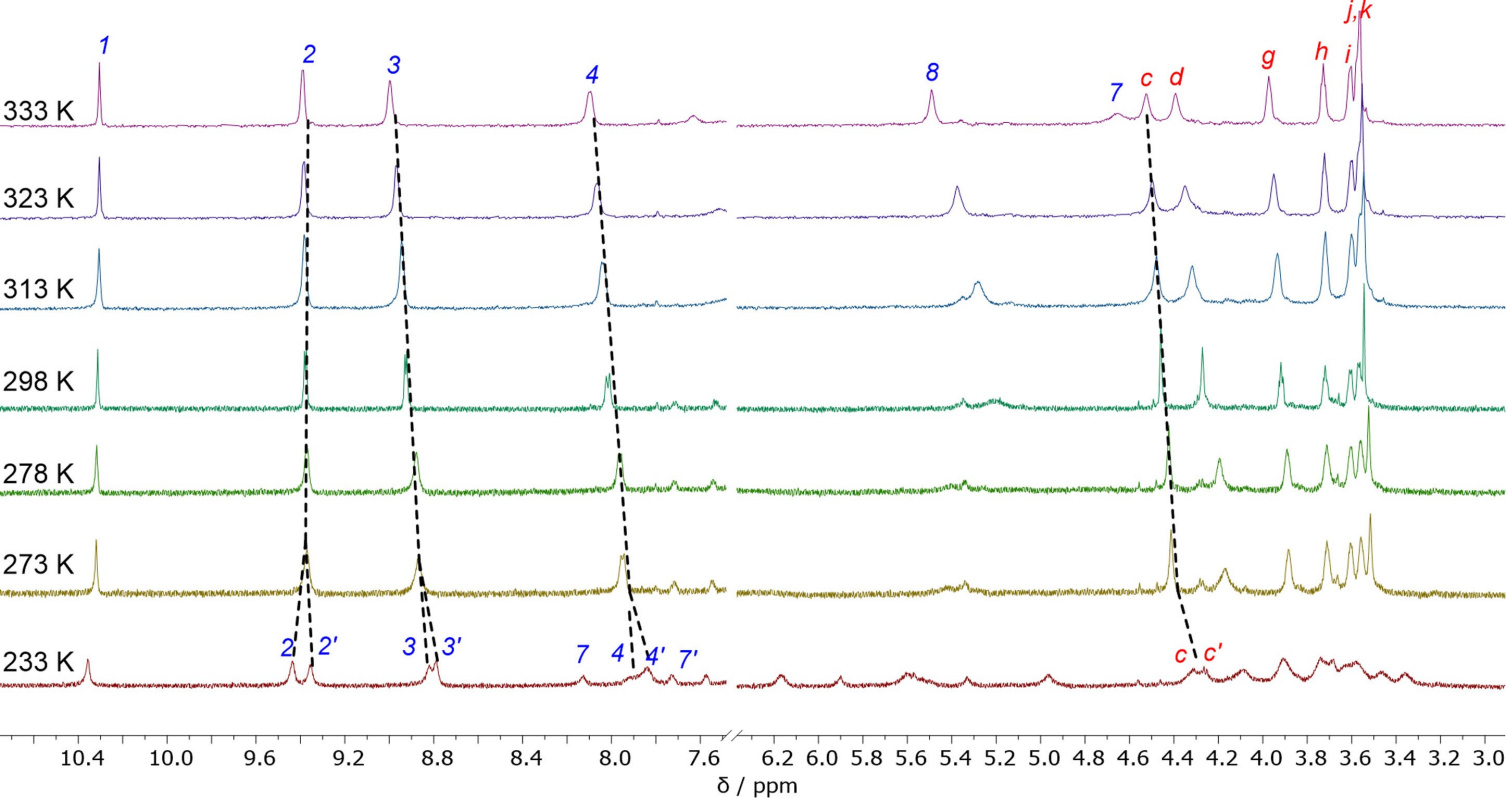
Stacked ^1^H NMR Spectra (500 MHz, CDCl_3_) of *p*‐**6 ⋅ Zn** recorded at temperatures in the range 233 K–333 K.

Variable temperature ^1^H NMR spectroscopic studies of the [2]rotaxanes were undertaken in CDCl_3_ to investigate their dynamic properties. The ^1^H NMR spectra of the [2]rotaxanes were recorded across a temperature range of – 30 to + 50  °C. In all cases at lower temperatures the splitting of several proton signals, including axle porphyrin derived *H_2_* and *H_3_* and macrocycle *H_c_* environments, was observed, whilst at higher temperatures these signals coalesced and became increasingly resolved (Figure 4). These observations are consistent with the hypothesis that the [2]rotaxane adopts, at low temperatures, a co‐conformation in which the macrocycle resides on one side of the central porphyrin axle core, thereby de‐symmetrising the chemical environments of the axle component. At higher temperatures, macrocycle shuttling across the length of the axle is fast on the ^1^H NMR timescale.

**Figure 5:**
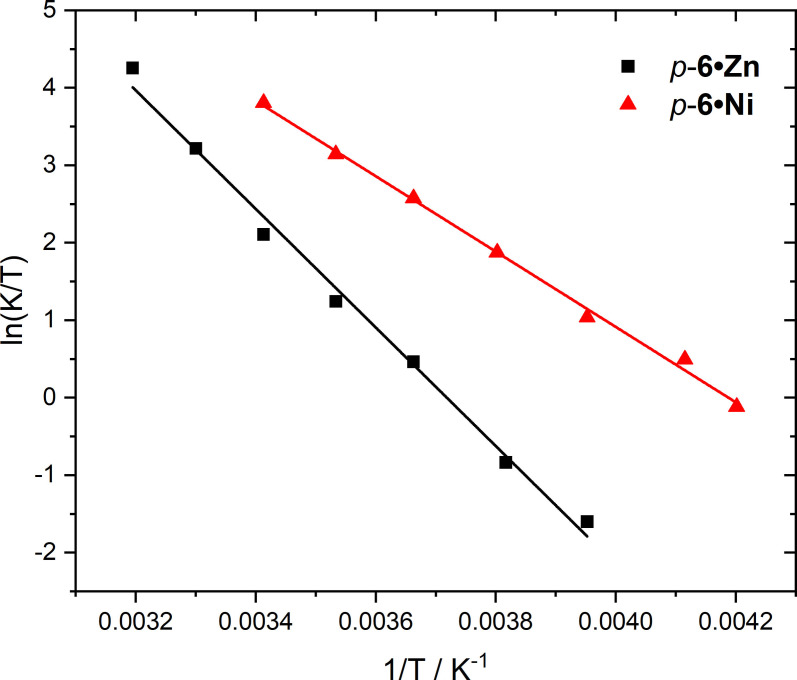
Eyring plots for *p*‐**6 ⋅ Zn** and *m‐* **6 ⋅ Zn** in CDCl_3_.

To elucidate the potential role of the Zn(II) centre in the dynamic properties of [2]rotaxane *p*‐**6 ⋅ Zn**, the free base porphyrin containing [2]rotaxane *p*‐**6 ⋅ H_2_** was prepared in excellent yield via a demetallation reaction using trifluoroacetic acid. A subsequent re‐metallation with Ni(acac)_2_ afforded *p*‐**6 ⋅ Ni** in 87 % yield (Scheme 1d). Exchange of the Zn(II) centre of *p*‐**6 ⋅ Zn** to *p*‐**6 ⋅ Ni** or demetallation to *p‐* **6 ⋅ H_2_**, was accompanied by a dramatic sharpening and resolution of the [2]rotaxane ^1^H NMR signals. This observation strongly suggests that the [2]rotaxane inter‐component macrocycle pyridyl⋅⋅⋅Zn(II) metalloporphyrin interaction restricts the macrocycle's shuttling motion across the axle in *p*‐**6 ⋅ Zn**. Quantitative determination of the [2]rotaxane shuttling rates by Eyring analysis (Figure 5) enabled the calculation of ΔH^ǂ^ and ΔS^ǂ^ values shown in Table 1. Inspection of the ΔH^ǂ^ values reveal the shuttling processes are universally endothermic with the largest ΔH^ǂ^ value observed for *p‐* **6 ⋅ Zn**, consistent with the loss of the strongest pyridyl⋅⋅⋅Zn(II) metalloporphyrin interaction upon shuttling. Interestingly, the determined ΔH^ǂ^ for *m* **‐5 ⋅ Zn** was substantially less endothermic, approaching the ΔH^ǂ^ of *p*‐**6 ⋅ Ni** and *p*‐**6 ⋅ H_2_**_,_ which have no substantial Lewis acidic properties (see below). This indicates a significantly diminished macrocycle‐axle intercomponent pyridyl⋅⋅⋅Zn(II) interaction with *m*‐**5 ⋅ Zn** in comparison to *p*‐**6 ⋅ Zn**, presumably due to unfavourable steric effects.

**Table 1:** Activation parameters for dynamic intramolecular macrocycle shuttling in [2]rotaxanes as determined by variable temperature ^1^H NMR spectroscopy (500 MHz, CDCl_3_). Numbers in parentheses represent calculated errors in activation parameter final digit.

	T_C_ [K]	ΔH^ǂ^ [kJ mol^−1^]	ΔS^ǂ^ [J K^−1^ mol^−1^]
*p*‐**6 ⋅ Zn**	262	61(2)	29(1)
*p*‐**6 ⋅ H_2_**	241	46(4)	−10(1)
*p*‐**6 ⋅ Ni**	238	42(1)	−21(1)
*m*‐**5 ⋅ Zn**	255	49(2)	−20(1)

The contrasting ΔS^ǂ^ values may be rationalised by taking account of the conformational freedom of the respective rotaxane. For *p*‐**6 ⋅ Ni** and *p*‐**6 ⋅ H_2_**, it is likely that the dominant contribution to the entropy of activation is the reduction in the conformational freedom of the macrocycle as it passes over the widest section of the axle's porphyrin motif. Such an effect was reported by Silvi and co‐workers,^[58]^ who observed a similar negative entropy of activation for the translocation of a dibenzo‐24‐crown‐8 macrocycle over an axle amide group which was attributed to the rigidification of the crown ether macrocycle.

In the case of *p*‐**6 ⋅ Zn**, shuttling of the macrocycle causes deviation from the dominant co‐conformation in which the metalloporphyrin is axially ligated by the macrocycle pyridyl nitrogen, leading to greater conformational freedom, and a positive ΔS^ǂ^. Indeed, the negative ΔS^ǂ^ value observed for *m*‐**5 ⋅ Zn**, with a differing axle geometry, demonstrates the interplay between various dynamic processes involved in macrocycle shuttling but is consistent with the weaker Zn(II)‐pyridyl interaction in the *meta* isomer.

**Figure 6:**
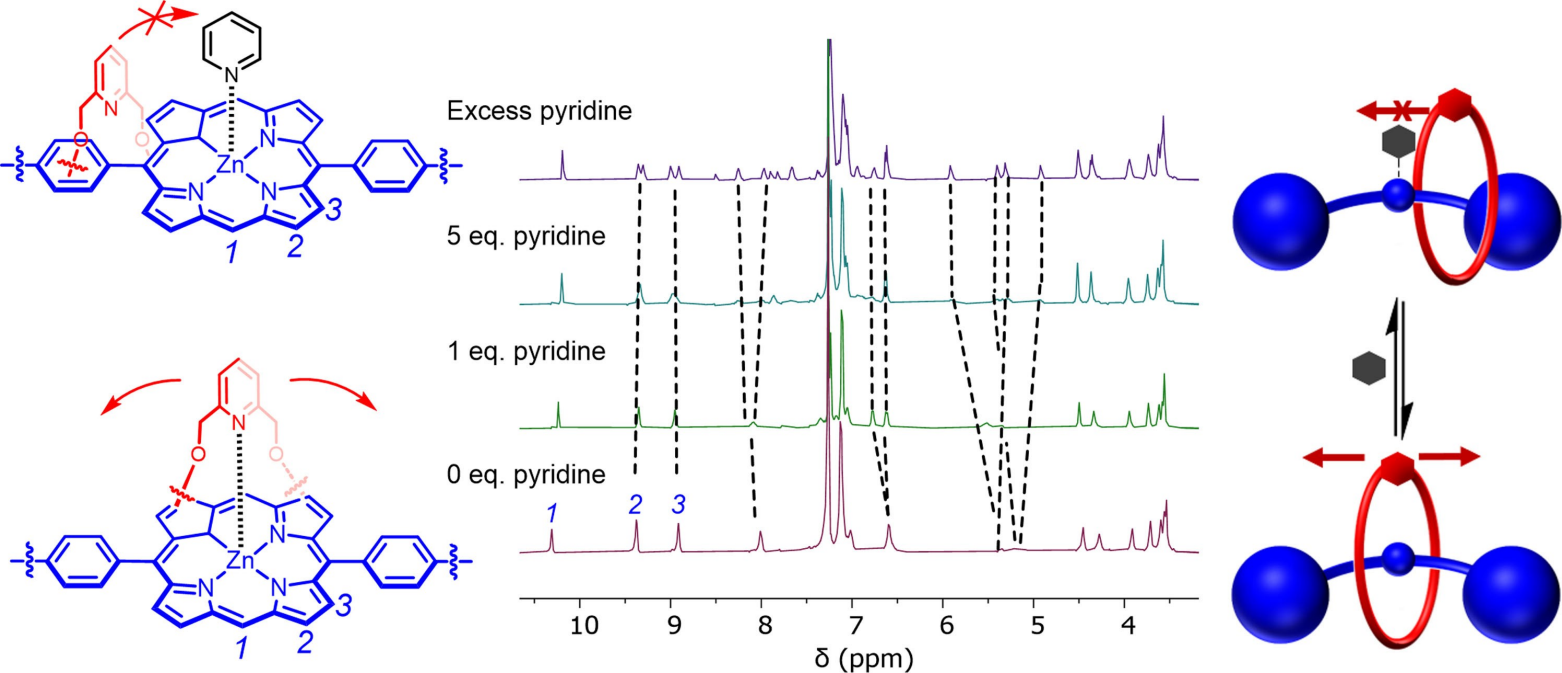
Stacked ^1^H NMR Spectra (400 MHz, CDCl_3_, 298 K) of *p*‐**6 ⋅ Zn** upon successive addition of pyridine.

It was of interest to investigate whether the addition of a competing Lewis base, such as pyridine, would affect the dynamic shuttling behaviour of rotaxane *p*‐**6 ⋅ Zn**. Upon adding increasing amounts of pyridine‐*d*_5_ to a CDCl_3_ solution of *p*‐**6 ⋅ Zn** the resolution of several broad proton signals associated with the axle and macrocycle‐based methylene environments improved and the splitting of these signals was observed (Figure 6). No equivalent behaviour was observed with analogous titration experiments conducted on the non‐interlocked axle. These results are consistent with macrocycle shuttling being prohibited by a sterically blocking ligand, leading to axle de‐symmetrisation.

**Figure 7:**
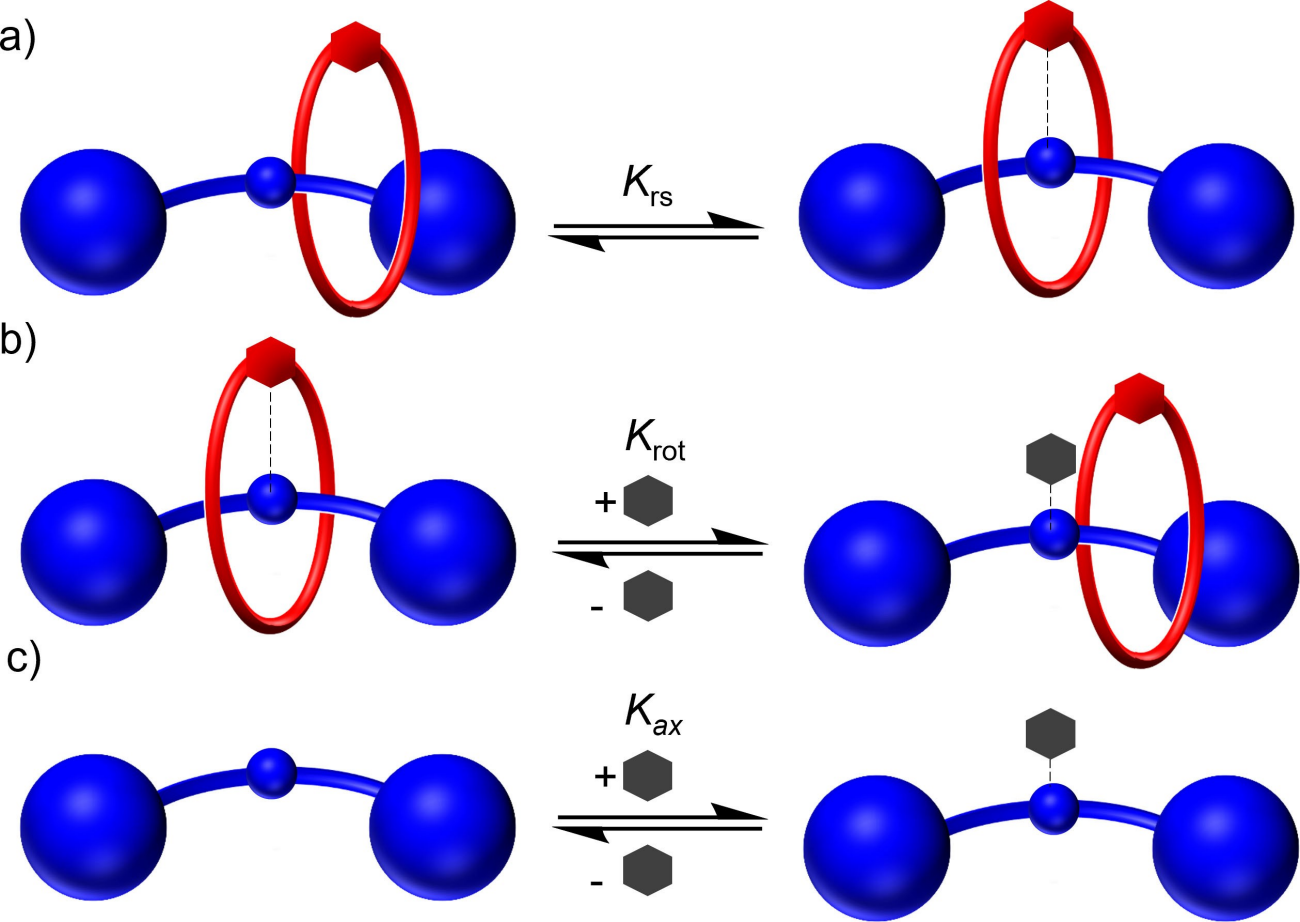
Cartoon depiction of the equilibria in Equation 1.

To further quantify the extent of this inter‐component pre‐organising interaction, quantitative UV‐visible spectroscopic titration experiments were conducted to determine the proportion of [2]rotaxanes existing in the inter‐component ligated ‘resting state’ conformation, at room temperature (represented in Figure 7a).^[58]^ The addition of pyridine to solutions of the [2]rotaxanes *m*‐**5 ⋅ Zn** and *p*‐**6 ⋅ Zn**, or the corresponding non‐interlocked axles *m*‐**9 ⋅ Zn** and *p*‐**10 ⋅ Zn**, effected bathochromic shifts in the porphyrin Soret‐band absorptions. Fitting of the resulting binding isotherms to a 1 : 1 host–guest stoichiometry using Bindfit^[59]^ determined binding constants of pyridine to both the rotaxanes and the non‐interlocked axle (*K*_rot_ and *K*_ax_, Figure 7 b and c, respectively). Knowledge of both binding constants enabled the calculation of the equilibrium constant for formation of the ‘resting state’ in accordance with Equation (1) (Table 2),^54^ and therefore the determination of the % of the rotaxane existing in the ‘resting state’ co‐conformation.

**Table 2:** Binding constants and %self‐inclusion calculated from titration of 25 mM pyridine solution with 7.5 μM *m*‐**5 ⋅ Zn** and *p‐* **6 ⋅ Zn** in CHCl_3_. Numbers in parentheses represent error in final digit. Errors for *K*_rs_ and resting state conformation propagated from calculated errors in experimental binding constants.

	*K*_ax_ [M^−1^]	*K*_rot_ [M^−1^]	*K*_rs_	% ‘resting state’ conformation
*m‐* **5 ⋅ Zn**	5660(470)	4810(420)	0.18(2)	15(3)
*p‐* **6 ⋅ Zn**	7630(290)	2850(300)	1.7(2)	63(9)

As expected from the earlier VT NMR studies, calculation of the percentage of [2]rotaxane in the ‘resting state’ co‐conformation reveals significant inter‐component interaction in *p*‐**6 ⋅ Zn**, with a calculated ‘resting state’ occupancy of 63 %, and a reduced interaction affording 15 % occupancy in *m*‐**5 ⋅ Zn**. This clear difference in co‐conformational bias is consistent with the different activation enthalpies for shuttling, as elucidated by variable‐temperature NMR. Accordingly, the significantly higher ΔH^ǂ^ for *p*‐**6 ⋅ Zn** is attributed to the enthalpic penalty of disrupting the ‘resting state’. The significantly lower ΔH^ǂ^ for *m*‐**5 ⋅ Zn**, is concordant with the lower enthalpic penalty of disrupting a weaker inter‐component interaction. It is notable that ΔH^ǂ^ for *m*‐**5 ⋅ Zn** approaches that of *p*‐**6 ⋅ H_2_**, in which no inter‐component interaction occurs and hence negligible bias for the ‘resting state’ is observed.

(1) $$K_{rs}=\frac{K_{ax}}{K_{rot}}-1$$

where $K_{ax}$ is the binding constant for pyridine to the axle, and $K_{rot}$ for pyridine to the [2]rotaxane (Figure 7).

   

**Figure 8:**
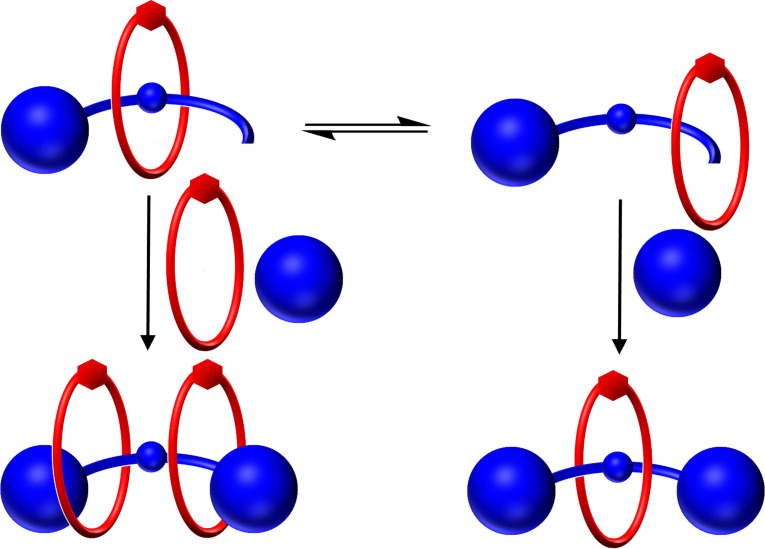
Cartoon depiction of pathways to [2] and [3]rotaxane formation during AMT reactions.

With convincing evidence for the presence of a strong inter‐component pyridyl⋅⋅⋅Zn(II) interaction in *p*‐**6 ⋅ Zn**, the significant higher order [3]rotaxane *p*‐**8 ⋅ Zn** product yield of 24 % (Scheme 1c) may be tentatively rationalised by a pseudo[2]rotaxane intermediate in which one of the two CuAAC stoppering reactions has been catalysed through the macrocycle interior. The strength of the inter‐component pyridyl⋅⋅⋅Zn(II) interaction retards macrocycle translocation, trapping the pseudo[2]rotaxane such that the second and final triazole forming stoppering reaction is completed by a separate Cu(I)‐complexed macrocycle, resulting in [3]rotaxane *p*‐**8 ⋅ Zn** formation (Figure 8).[^51^, ^59^]

### Anion binding studies

We next sought to exploit the interruption of the rotaxane inter‐component interaction, via an anion coordinating stimulus wherein competing halide anions induce macrocycle translocation, resulting potentially in an optical response.

**Figure 9:**
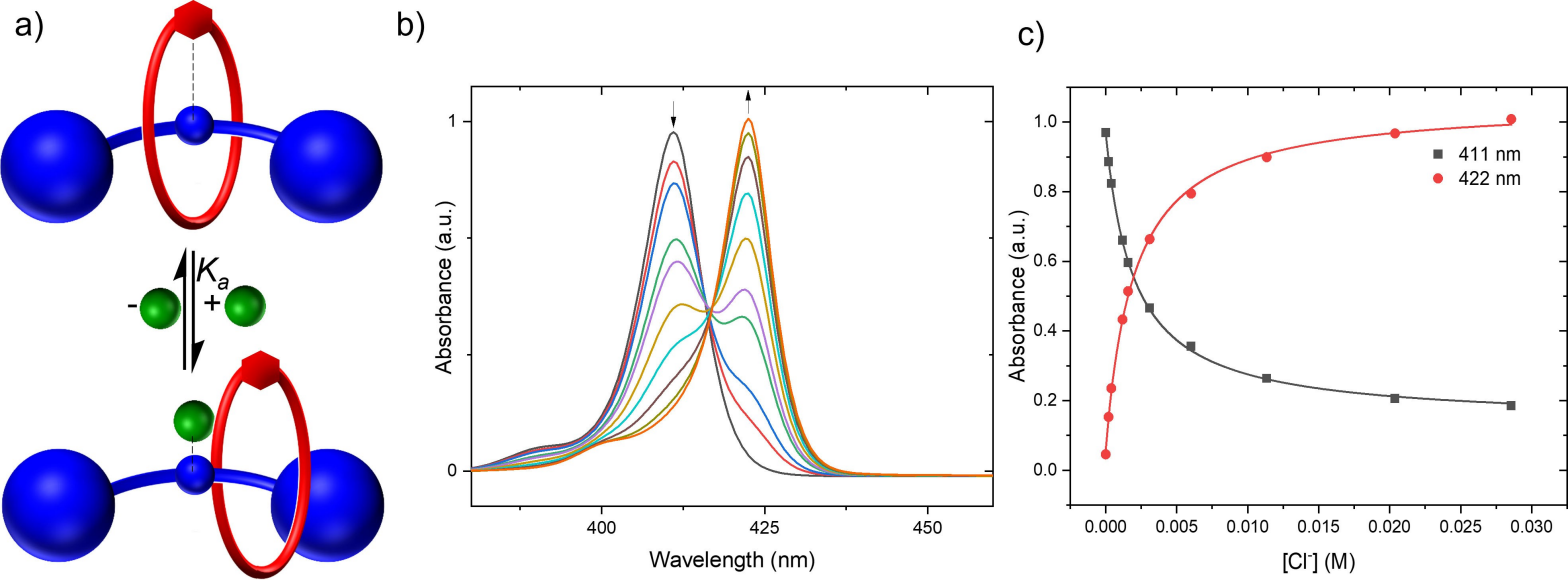
a) Cartoon depiction of anion binding mode of rotaxanes, b) Soret‐ and Q‐band region absorption spectrum of a 2 μM acetone solution of *p*‐**6 ⋅ Zn** upon successive addition of a TBACl solution in acetone. Black arrows indicate direction of change. c) Binding isotherms for the data shown in a).

To this end, UV‐visible spectroscopic halide titration experiments were performed on rotaxanes, *m*‐**5 ⋅ Zn**, *p*‐**6 ⋅ Zn**, *p*‐**6 ⋅ Ni**, *p*‐**6 ⋅ H_2_**_,_ *m*‐**7 ⋅ Zn** and *p*‐**8 ⋅ Zn**, and the non‐interlocked axles *m*‐**9 ⋅ Zn** and *p*‐**10 ⋅ Zn**. Typically, successive additions of the *tert*‐butyl ammonium (TBA) halide salts, TBAX, X=Cl, Br, to 2 μM acetone solutions of the hosts resulted in significant perturbation in the Soret and Q bands of the UV‐visible spectra of hosts containing a Zn(II) metalloporphyrin centre with bathochromic shifts greater than 10 nm observed (Figure 9b).^[60]^ A clear isosbestic point was observed in all cases, indicating a 1 : 1 host:guest binding stoichiometry. No such perturbations were observed for iodide, which may be rationalised by the lower charge density, and hence lower Lewis basicity, of the halide anion. Binding isotherms were plotted from the intensity of the Soret band absorption (Figure 9 c) and 1 : 1 host:guest association constants were determined by Bindfit analysis (Table 3).^[61]^ The trend in determined anion association constants mirrors that of halide charge density, namely Cl^−^>Br^−^>I^−^.^[39]^ As expected, in all cases the binding constants for the [2]rotaxanes are significantly reduced over those for the non‐interlocked axles. This phenomenon is attributed to the competing rotaxane co‐ordination at the Zn(II) metalloporphyrin centre by the macrocycle pyridyl group. The lower chloride anion binding constant for [3]rotaxane *p*‐**8 ⋅ Zn** compared with *p*‐**6 ⋅ Zn**, may be attributed to the increased effective concentration of the competing pyridyl ligand when two macrocycles are present in the higher order interlocked system. Likewise, this increase in local pyridyl concentration may account for the *K*_a_ (Cl^−^) trend *m*‐**5 ⋅ Zn**>*m*‐**7 ⋅ Zn**. No binding was observed upon the addition of any halide tested to *p‐* **6 ⋅ H_2_** nor to *p‐* **6 ⋅ Ni**, consistent with the lack of Lewis acidic centre in the free base or Ni(II) [2]rotaxanes.

**Table 3:** Host–guest binding constants for 2 μM indicated host and 100 mM TBAX, X=Cl, Br, I solutions in acetone. Deviations in Soret‐band absorption upon binding fitted to a 1 : 1 host:guest stoichiometry. Numbers in parentheses represent error in final digit.^[a]^

	*K*_a_ [M^−1^]							
	*p*‐**6 ⋅ Zn**	*p*‐**8 ⋅ Zn**	*p*‐**10 ⋅ Zn**	*m*‐**5 ⋅ Zn**	*m*‐**7 ⋅ Zn**	*m*‐**9 ⋅ Zn**	*p*‐**6 ⋅ H_2_**	*p*‐**6 ⋅ Ni**
Cl^−^	660(40)	550(30)	910(45)	840(40)	750(40)	970(45)	NB	NB
Br^−^	40(3)	35(3)	40(3)	40(3)	30(3)	40(4)	NB	NB
I^−^	NB	NB	NB	NB	NB	NB	NB	NB

[a] NB=no binding observed.

The reduction in chloride binding constant observed between the [2]rotaxane and corresponding non‐interlocked axles was significantly less for *m*‐**5 ⋅ Zn** and *m*‐**9 ⋅ Zn** than the regioisomeric analogues *p*‐**6 ⋅ Zn** and *p*‐**10 ⋅ Zn**. The relative similarity of the chloride binding constant values between axle isomers *m*‐**9 ⋅ Zn** and *p*‐**10 ⋅ Zn**, strongly suggests the difference is not the result of a changed mode of axle halide binding, nor due to significant changes in sterics upon changing the linker. It is more likely due to the diminished prevalence of the ‘resting state’ in *m*‐**5 ⋅ Zn**, and hence reduced competition for the vacant axial co‐ordination site of the Zn(II) metalloporphyrin. Hence, rotaxane host‐Cl‐guest binding induces an optical response which is directly correlated to the inter‐component interaction afforded by the mechanical bond.

## Conclusions

In summary, a series of dynamic metalloporphyrin axle containing [2]‐ and [3]‐rotaxanes were prepared via AMT mechanical bond synthesis in high yields, wherein the nature of the Lewis acidic metallocentre dictates the strength of inter‐component axle‐macrocycle shuttling behaviour and halide anion co‐ordinating capability.

The rotaxanes contained either Zn(II), Ni(II) metallo‐, or free‐base porphyrin axle and pyridyl functionalised macrocycle components. Variable temperature ^1^H NMR and UV‐Vis spectroscopic studies demonstrated the [2]rotaxane macrocycle translocation or shuttling behaviour is principally governed by the Lewis acidity of the porphyrin metal centre. Specifically, the macrocycle pyridyl co‐ordination interaction with the Zn(II) centre of the axle porphyrin, in the ‘resting state’ co‐conformation. The resting state was disrupted by addition of a competing neutral Lewis base pyridine or by halide anions, both of which stimulate dynamic shuttling behaviour via co‐ordination to Zn(II) and displacement of the macrocycle.

Importantly, Lewis base pyridine or in particular halide binding to the Zn(II) metalloporphyrin centre was accompanied by marked isosbestic changes to the porphyrin Soret absorption band, affording an optical sensory response that signals mechanical bond dynamic shuttling and anion detection.

## Conflict of interest

There are no conflicts to declare.

1

## Supporting information

As a service to our authors and readers, this journal provides supporting information supplied by the authors. Such materials are peer reviewed and may be re‐organized for online delivery, but are not copy‐edited or typeset. Technical support issues arising from supporting information (other than missing files) should be addressed to the authors.

Supporting Information

## Data Availability

The data that support the findings of this study are available in the supplementary material of this article.

## References

[1] J. R. Romero , G. Aragay , P. Ballester , Chem. Sci. 2017, 8, 491–498. 28451196 10.1039/c6sc03554j PMC5341206

[2] T. Bunchuay , A. Docker , A. J. Martinez-Martinez , P. D. Beer , Angew. Chem. Int. Ed. 2019, 58, 13823–13827; 10.1002/anie.201907625 31291498

[3] M. Cirulli , A. Kaur , J. E. M. Lewis , Z. Zhang , J. A. Kitchen , S. M. Goldup , M. M. Roessler , J. Am. Chem. Soc. 2019, 141, 879–889. 30562470 10.1021/jacs.8b09715

[4] M. Nandi , S. Bej , T. K. Ghosh , P. Ghosh , Chem. Commun. 2019, 55, 3085–3088. 10.1039/c9cc00090a 30785460

[5] A. Docker , Y. C. Tse , H. M. Tay , A. J. Taylor , Z. Zhang , P. D. Beer , Angew. Chem. Int. Ed. 2022, 134, e202214523. 10.1002/anie.202214523 PMC10100147 36264711

[6] R. J. Goodwin , A. Docker , H. I. MacDermott-Opeskin , H. M. Aitken , M. L. O′Mara , P. D. Beer , N. G. White , Chem. Eur. J. 2022, 28, e202200389. 35293643 10.1002/chem.202200389 PMC9321576

[7] K. M. Bąk , K. Porfyrakis , J. J. Davis , P. D. Beer , Mater. Chem. Front. 2020, 4, 1052–1073.

[8] M. Denis , J. Pancholi , K. Jobe , M. Watkinson , S. M. Goldup , Angew. Chem. Int. Ed. 2018, 57, 5310–5314; 10.1002/anie.201712931 PMC5947674 29537728

[9] M. Denis , L. Qin , P. Turner , K. A. Jolliffe , S. M. Goldup , Angew. Chem. Int. Ed. 2018, 57, 5315–5319; 10.1002/anie.201713105 PMC5947583 29393993

[10] S.-M. Chan , F.-K. Tang , C.-S. Kwan , C.-Y. Lam , S. C. K. Hau , K. C.-F. Leung , Mater. Chem. Front. 2019, 3, 2388–2396.

[11] A. Martinez-Cuezva , A. Saura-Sanmartin , M. Alajarin , J. Berna , ACS Catal. 2020, 10, 7719–7733.

[12] Y.-J. Lee , K.-S. Liu , C.-C. Lai , Y.-H. Liu , S.-M. Peng , R. P. Cheng , S.-H. Chiu , Chem. Eur. J. 2017, 23, 9756–9760. 28577323 10.1002/chem.201702525

[13] M. Galli , J. E. M. Lewis , S. M. Goldup , Angew. Chem. Int. Ed. 2015, 54, 13545–13549; 10.1002/anie.201505464 PMC4678423 26387887

[14] J. Y. C. Lim , N. Yuntawattana , P. D. Beer , C. K. Williams , Angew. Chem. Int. Ed. 2019, 58, 6007–6011; 10.1002/anie.201901592 PMC6519244 30861303

[15] J. Lewis , M. Galli , S. Goldup , Chem. Commun. 2017, 53, 298–312. 10.1039/c6cc07377h 27819362

[16] E. A. Neal , S. M. Goldup , Chem. Commun. 2014, 50, 5128–5142. 10.1039/c3cc47842d 24434901

[17] J. E. M. Lewis , M. Galli , S. M. Goldup , Chem. Commun. 2017, 53, 298–312. 10.1039/c6cc07377h 27819362

[18] P. L. Anelli , N. Spencer , J. F. Stoddart , J. Am. Chem. Soc. 1991, 113, 5131–5133. 27715028 10.1021/ja00013a096

[19] K. M. Mullen , J. Mercurio , C. J. Serpell , P. D. Beer , Angew. Chem. Int. Ed. 2009, 48, 4781–4784; 10.1002/anie.200901313 19452507

[20] T. A. Barendt , A. Docker , I. Marques , V. Félix , P. D. Beer , Angew. Chem. Int. Ed. 2016, 55, 11069–11076; 10.1002/anie.201604327 PMC5113793 27436297

[21] J. Berná , M. Alajarín , R.-A. Orenes , J. Am. Chem. Soc. 2010, 132, 10741–10747. 20681706 10.1021/ja101151t

[22] C. Gao , Z.-L. Luan , Q. Zhang , S. Yang , S.-J. Rao , D.-H. Qu , H. Tian , Org. Lett. 2017, 19, 1618–1621. 28304173 10.1021/acs.orglett.7b00393

[23] N. H. Evans , C. J. Serpell , P. D. Beer , Chem. Commun. 2011, 47, 8775–8777. 10.1039/c1cc13247d 21743929

[24] T. Iijima , S. A. Vignon , H.-R. Tseng , T. Jarrosson , J. K. M. Sanders , F. Marchioni , M. Venturi , E. Apostoli , V. Balzani , J. F. Stoddart , Chem. Eur. J. 2004, 10, 6375–6392. 15532018 10.1002/chem.200400651

[25] G. T. Spence , M. B. Pitak , P. D. Beer , Chem. Eur. J. 2012, 18, 7100–7108. 22550020 10.1002/chem.201200317

[26] T. A. Barendt , L. Ferreira , I. Marques , V. Félix , P. D. Beer , J. Am. Chem. Soc. 2017, 139, 9026–9037. 28590726 10.1021/jacs.7b04295

[27] H. A. Klein , H. Kuhn , P. D. Beer , Chem. Commun. 2019, 55, 9975–9978. 10.1039/c9cc04752b 31367706

[28] C.-F. Lin , C.-C. Lai , Y.-H. Liu , S.-M. Peng , S.-H. Chiu , Chem. Eur. J. 2007, 13, 4350–4355. 17323386 10.1002/chem.200601432

[29] C. J. Serpell , R. Chall , A. L. Thompson , P. D. Beer , Dalton Trans. 2011, 40, 12052–12055. 21445380 10.1039/c1dt10186b

[30] M. J. Barrell , D. A. Leigh , P. J. Lusby , A. M. Z. Slawin , Angew. Chem. Int. Ed. 2008, 47, 8036–8039; 10.1002/anie.200802745 18792909

[31] T. A. Barendt , I. Rašović , M. A. Lebedeva , G. A. Farrow , A. Auty , D. Chekulaev , I. V. Sazanovich , J. A. Weinstein , K. Porfyrakis , P. D. Beer , J. Am. Chem. Soc. 2018, 140, 1924–1936. 29337535 10.1021/jacs.7b12819

[32] H. M. Tay , Y. C. Tse , A. Docker , C. Gateley , A. L. Thompson , H. Kuhn , Z. Zhang , P. D. Beer , Angew. Chem. Int. Ed. 2023, 62, e2022147. 10.1002/anie.202214785 PMC10108176 36440816

[33] Y. Cheong Tse , R. Hein , E. J. Mitchell , Z. Zhang , P. D. Beer , Chem. Eur. J. 2021, 27, 14550–14559. 34319624 10.1002/chem.202102493 PMC8596797

[34] Y.-L. Huang , W.-C. Hung , C.-C. Lai , Y.-H. Liu , S.-M. Peng , S.-H. Chiu , Angew. Chem. Int. Ed. 2007, 46, 6629–6633; 10.1002/anie.200702197 17665407

[35] K. M. Mullen , J. J. Davis , P. D. Beer , New J. Chem. 2009, 33, 769–776.

[36] D. P. Cormode , M. G. B. Drew , R. Jagessar , P. D. Beer , Dalton Trans. 2008, 6732–6741. 19153621 10.1039/b807153e

[37] D. P. Cormode , S. S. Murray , A. R. Cowley , P. D. Beer , Dalton Trans. 2006, 5135–5140. 17077886 10.1039/b609817g

[38] L. C. Gilday , N. G. White , P. D. Beer , Dalton Trans. 2013, 42, 15766–15773. 24056495 10.1039/c3dt52093e

[39] L. C. Gilday , N. G. White , P. D. Beer , Dalton Trans. 2012, 41, 7092–7097. 22561990 10.1039/c2dt30124e

[40] K.-I. Hong , H. Yoon , W.-D. Jang , Chem. Commun. 2015, 51, 7486–7488. 10.1039/c5cc00809c 25831467

[41] J. B. Allison, R. S. Becker, **1960**, *J. Chem. Phys*., *32*, 1410–1417.

[42] A. Antipas , D. Dolphin , M. Gouterman , E. C. Johnson , J. Am. Chem. Soc. 1978, 100, 7705–7709.

[43] R. S. Becker , J. B. Allison , J. Phys. Chem. 1963, 67, 2662–2669.

[44] T. Mizutani , T. Kurahashi , T. Murakami , N. Matsumi , H. Ogoshi , J. Am. Chem. Soc. 1997, 119, 8991–9001.

[45] C. Lee , D. H. Lee , J.-I. Hong , Tetrahedron Lett. 2001, 42, 8665–8668.

[46] M. Wolf , A. Ogawa , M. Bechtold , M. Vonesch , J. A. Wytko , K. Oohora , S. Campidelli , T. Hayashi , D. M. Guldi , J. Weiss , Chem. Sci. 2019, 10, 3846–3853. 30996970 10.1039/c8sc05328f PMC6446966

[47] X. Ma , J. Zhang , J. Cao , X. Yao , T. Cao , Y. Gong , C. Zhao , H. Tian , Chem. Sci. 2016, 7, 4582–4588. 30155105 10.1039/c6sc00769d PMC6016324

[48] S. M. Goldup , D. A. Leigh , P. J. Lusby , R. T. McBurney , A. M. Z. Slawin , Angew. Chem. Int. Ed. 2008, 47, 3381–3384; 10.1002/anie.200705859 18357597

[49] J. Berná , S. M. Goldup , A.-L. Lee , D. A. Leigh , M. D. Symes , G. Teobaldi , F. Zerbetto , Angew. Chem. Int. Ed. 2008, 47, 4392–4396; 10.1002/anie.200800891 18442159

[50] S. M. Goldup , D. A. Leigh , T. Long , P. R. McGonigal , M. D. Symes , J. Wu , J. Am. Chem. Soc. 2009, 131, 15924–15929. 19807083 10.1021/ja9070317

[51] V. Aucagne , J. Berná , J. D. Crowley , S. M. Goldup , K. D. Hänni , D. A. Leigh , P. J. Lusby , V. E. Ronaldson , A. M. Z. Slawin , A. Viterisi , D. B. Walker , J. Am. Chem. Soc. 2007, 129, 11950–11963. 17845039 10.1021/ja073513f

[52] D. A. Roberts , T. W. Schmidt , M. J. Crossley , S. Perrier , Chem. Eur. J. 2013, 19, 12759–12770. 23939811 10.1002/chem.201301133

[53] A. Tron , P. J. Thornton , M. Rocher , H.-P. Jacquot de Rouville , J.-P. Desvergne , B. Kauffmann , T. Buffeteau , D. Cavagnat , J. H. R. Tucker , N. D. McClenaghan , Org. Lett. 2014, 16, 1358–1361. 24571171 10.1021/ol500099u

[54] The possibility of the postulated binding interaction arising through the oxygen atoms of the macrocycle PEG chain rather than the pyridyl moiety was also considered, given the documented low nucleophilicity of 2,6-lutidine derivatives. To this end Soret band UV-visible absorption and ^1^H NMR spectroscopic titrations of 2,6-lutidine with *p*-**6 ⋅ Zn** were performed (see Supporting Information), in both cases spectral perturbations strongly indicate the ability of the 2,6-substituted pyridines to intract with the rotaxane Zn(II) core, thus we are confident in the interaction of a favourable interaction between the macrocycle pyridyl and the metalloporphyrin. Given previous reports of binding constants for similarly methyl substituted pyridines binding more strongly to Zn(II) tetraphenyl porphyrin than ether oxygen donors, binding of the nitrogenous base to the Zn(II) core is likely the dominant binding interaction between the macrocycle and the axle core.^[62,63]^.

[55] S. W. Hewson , K. M. Mullen , Org. Biomol. Chem. 2018, 16, 8569–8578. 30375613 10.1039/c8ob02304b

[56] M. Nappa , J. S. Valentine , J. Am. Chem. Soc. 1978, 100, 5075–5080.

[57] L. Favereau , A. Cnossen , J. B. Kelber , J. Q. Gong , R. M. Oetterli , J. Cremers , L. M. Herz , H. L. Anderson , J. Am. Chem. Soc. 2015, 137, 14256–14259. 26536147 10.1021/jacs.5b10126 PMC4686216

[58] S. Corra , C. de Vet , M. Baroncini , A. Credi , S. Silvi , Chem 2021, 7, 2137–2150. 34435161 10.1016/j.chempr.2021.04.010 PMC8367298

[59] E. A. Neal , S. M. Goldup , Chem. Sci. 2015, 6, 2398–2404. 29308153 10.1039/c4sc03999h PMC5645920

[60] The reversibility of the shuttling-induced bathochromic shift was demonstrated by addition of AgOTf to the titration solution of *p*-**6 ⋅ Zn** and TBACl at the end point. Precipitation of the corresponding silver halide salt was observed and the Soret band absorption maximum returned to the original, free *p*-**6 ⋅ Zn**, value. (Figure S31.).

[61] P. Thordarson , Chem. Soc. Rev. 2011, 40, 1305–1323. 21125111 10.1039/c0cs00062k

[62] J. V. Nardo , J. H. Dawson , Inorg. Chim. Acta 1986, 123, 9–13.

[63] C. H. Kirksey , P. Hambright , C. B. Storm , Inorg. Chem. 1969, 8, 2141–2144.

