# Supplementary material for: Dynamic Metalloporphyrin‐Based [2]Rotaxane Molecular Shuttles Stimulated by Neutral Lewis Base and Anion Coordination
Source: Chemistry. 2023 Apr 26;29(33):e202300608. doi: 10.1002/chem.202300608 (PMC10947143; doi:10.1002/chem.202300608)
Supplement: Supplementary file 1 — Supporting Information [file CHEM-29-0-s001.pdf]

Chemistry–A European Journal

Supporting Information

Dynamic Metalloporphyrin-Based [2]Rotaxane Molecular Shuttles Stimulated by Neutral Lewis Base and Anion Coordination

Jamie T. Wilmore, Yuen Cheong Tse, Andrew Docker, Caspar Whitehead, Charlotte K. Williams, and Paul D. Beer*

Table of Contents

S1 General Experimental Procedures S2

S2 Novel Compound Synthesis S3

S3 Spectral Characterisation of Novel Structures S12

S4 2,6-Lutidine Binding Studies S23

S5 Variable Temperature NMR Studies S24

S6 Measurement of the ‘Resting State’ Bias S25

S7 Binding Studies of Pyridine to [3]Rotaxane S25

S8 Optical Titration Studies of TBAX Salt Anion Binding S26

S9 Reversibility of TBAX Salt Anion Binding S27

S10 References S28

S1 General Experimental Procedures

Solvents and reagents were purchased from commercial suppliers and used as received. Dry solvents were obtained by purging with nitrogen and passing through a MBraun MPSP-800 column. H_2O was de-ionised and micro-filtered using a Milli-Q® Millipore machine.

Experiments were conducted at room temperature unless otherwise stated. Merck silica gel 60 was used for flash column chromatography. TBA salts were stored in vacuum desiccators prior to use. NMR spectra were either recorded on a Bruker Avance III HD Nanobay NMR spectrometer equipped with a 9.4 T magnet or a Bruker NEO 600 with broadband helium cryoprobe. ^{1}H NMR titrations were recorded on a Bruker Avance III NMR equipped with a 11.75 T magnet.

Chemical shifts are quoted in parts per million relative to the residual solvent peak. UV-visible titration anion binding experiments were performed at 298 K using a Horiba Duetta. Triethylamine was distilled from and stored over potassium hydroxide. Tris[(1-benzyl-1H-1,2,3-triazol-4-yl)methyl]amine (TBTA).

The following compounds were prepared according to literature procedures: **1**,[1] *p*-**4•H$_2$**[2] and *p*-**4•Zn**.[2]

S2 Novel Compound Synthesis

<u>Macrocycle **2**</u>

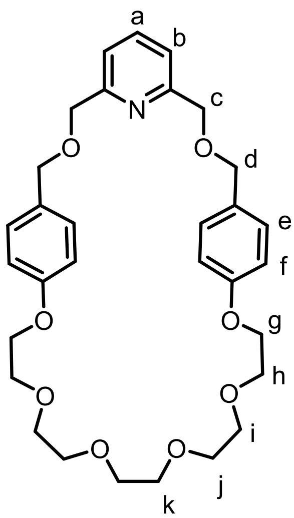

2

NaH (0.80 g, 19.92 mmol, 60 % w/w dispersion) was suspended in anhydrous THF (100 ml) and stirred at 70°C for 30 minutes. Separate solutions of 2,6-bis(bromomethyl)pyridine (1.19 g, 4.98 mmol) and the diol **1** (2.03 g, 4.98 mmol), each in THF (50 ml), were added dropwise to the refluxing solution over the course of 3 hours. Once the addition was complete, the mixture was left to stir overnight at 70°C. After which, the mixture was left to cool to room temperature and was carefully quenched by the addition of MeOH (50 ml). The resultant mixture was concentrated to dryness *in vacuo* and the crude reaction mixture was partitioned between DCM (250 ml) and H_2O (250 ml), the organic phase was collected and washed with H_2O (250 ml), dried over $MgSO_4$ and the solvent removed *in vacuo*. The crude reaction mixture was purified by column chromatography (3:2:0.3 DCM:EtOAc:MeOH *v/v*) to afford the product. Yield: 1.05 g (38%).

^{1}H NMR (500 MHz, $CDCl_3$, 298 K) δ: 7.67 (t, J = 7.7 Hz, 1H, H_a), 7.33 (d, J = 7.7 Hz, 2H, H_b), 7.23 (d, J = 8.6 Hz, 4H, H_e), 6.83 (d, J = 8.6 Hz, 4H, H_f), 4.57 (s, 4H, H_c), 4.50 (s, 4H, H_d), 4.09 (dd, J = 5.6, 4.0 Hz, 4H, H_g), 3.83 (dd, J = 5.7, 4.0 Hz, 4H, H_h), 3.73 – 3.63 (m, 12H,) ppm.
^{13}C{^{1}H} NMR (126 MHz, $CDCl_3$, 298 K) δ: 158.5, 157.8, 137.0, 130.0, 129.9, 120.5, 114.5, 72.0, 71.8, 71.0, 70.8, 70.7, 69.6, 67.6 ppm.
HR ESI-MS m/z calc'd for $[C_{31}H_{40}NO_8]^+$, $[M+H]^+$, 554.2748, found: 554.2747.

Free base *meta*-Functionalised Porphyrin, **3•H2**

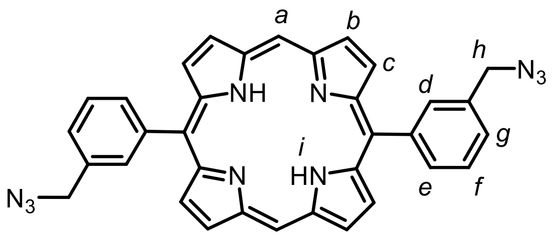

m-**3•H2**

3-(azidomethyl)benzaldehyde (1.0 g, 6.21 mmol) and dipyrrolemethane (0.91 g, 6.21 mmol) were dissolved in dry DCM (1200 mL) and the solution degassed under exclusion of light for 30 min. Trifluoroacetic acid (0.36 mL, 4.72 mmol) was added dropwise and the reaction mixture stirred at room temperature under N_2 for 24 h. 2,3-Dichloro-5,6-dicyano-*p*-benzoquinone (2.11 g, 9.31 mmol) was added as a solid, and the mixture stirred for 2 h, before dropwise addition of triethylamine (0.87 mL, 6.21 mmol). The reaction mixture was concentrated *in vacuo* and loaded onto a silica pad. The crude product was eluted with DCM (500 mL) until the solution ran clear. The purple solution was concentrated to 50 mL *in vacuo* and the product precipitated with MeOH as a purple crystalline solid. Yield: 0.889 g (50%).

^{1}H NMR (500 MHz, $CDCl_3$, 298 K) δ: 10.35 (s, 2H, H_a), 9.42 (d, J = 4.6 Hz, 4H, H_b), 9.07 (d, J = 4.6 Hz, 4H, H_c), 8.33 – 8.20 (m, 4H, $H_{d,e}$), 7.89 – 7.77 (m, 4H, $H_{f,g}$), 4.70 (s, 4H, H_h), -3.13 (s, 2H, H_i) ppm.
^{13}C{^{1}H} NMR (126 MHz, $CDCl_3$, 298 K) δ: 147.1, 145.3, 142.0, 134.8, 134.6, 134.2, 131.9, 130.9, 127.7, 127.6, 118.4, 105.5, 54.9 ppm.
HR ESI-MS m/z calc'd for $[C_{34}H_{25}N_{10}]^+$, $[M+H]^+$, 573.2258, found: 573.2257.

Zn(II) *meta*-Functionalised Metalloporphyrin, *m*-**3•Zn**

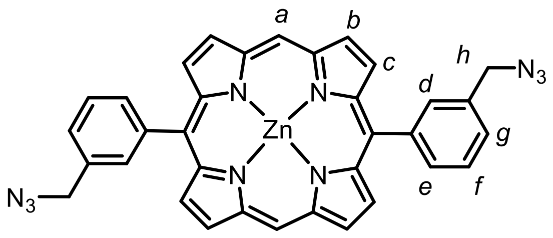

m-**3•Zn**

The free-base porphyrin *m*-**3•H2** (0.890 g, 1.55 mmol) was suspended in a 1:1 *v/v* mixture of DCM and MeOH (100 mL). $Zn(OAc)_2 \cdot 2H_2O$ (1.71 g, 7.95 mmol) was injected as a saturated methanolic solution. The resulting suspension was stirred under N_2 for 24 h, and the solvent removed *in vacuo* to afford a pink solid, which was taken up in DCM (200 mL) and washed

with water (3 x 100 mL). The organic layer was dried over $MgSO_4$ and the volatiles removed *in vacuo* to afford *m*-**3•Zn** as a pink solid which was used for subsequent synthesis without further purification. Yield: 988 mg (Quantitative).

^{1}H NMR (500 MHz, $CDCl_3$) δ: 10.28 (s, 2H, H_a), 9.39 (d, J = 4.5 Hz, 4H, H_b), 9.04 (d, J = 4.5 Hz, 4H, H_c), 8.18 (d, J = 7.4 Hz, 2H, H_e), 8.11 (s, 2H, H_d), 7.74 (t, J = 7.4 Hz, 2H, H_f), 7.68 (d, J = 7.4 Hz, 2H, H_g), 4.52 (s, 4H, H_h) ppm.

^{13}C NMR (126 MHz, $CDCl_3$) δ: 150.0, 149.6, 144.1, 134.9, 134.7, 133.5, 132.1, 131.8, 127.2, 127.0, 118.8, 106.0, 55.1 ppm.

HR ESI-MS m/z calc'd for $[C_{34}H_{23}N_{10}Zn]^+$, $[M+H]^+$, 635.1393, found: 635.1385.

<u>*Meta*-substituted Zn(II) Metalloporphyrin [2]- and [3]-Rotaxanes *m*-**5•Zn**, *m*-**7•Zn**</u>

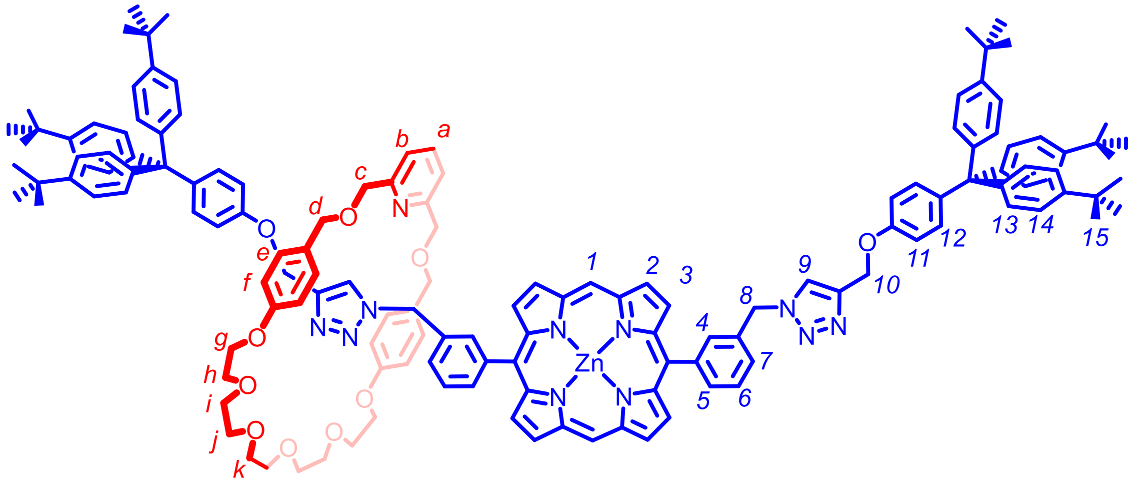

m-**5•Zn**

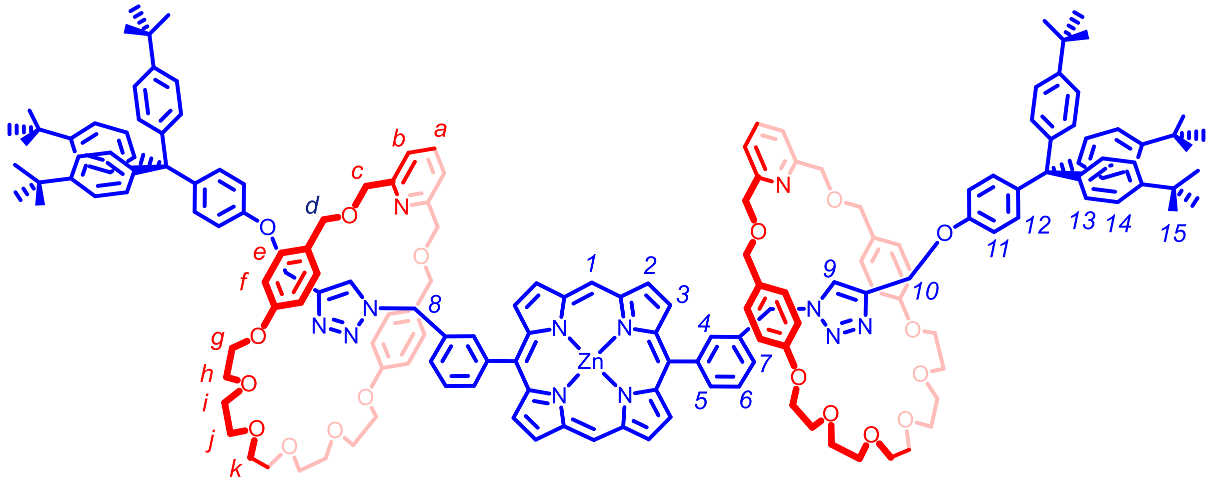

m-**7•Zn**

Macrocycle **2** (20.0 mg, 0.036 mmol) and $[Cu(CH_3CN)_4]PF_6$ (6.4 mg, 0.017 mmol) were added to dry, degassed CH_2ClCH_2Cl (DCE, 1.6 ml) in a sealed flask. The mixture was then purged with N_2 for 5 minutes before stirring at room temperature under N_2 for 15 minutes. A solution

of *m*-**3•Zn** (34.5 mg, 0.054 mmol) in dry, degassed DCE (1.0 ml) was added, followed by addition of a solution of stopper-alkyne[3] (59.0 mg, 0.108 mmol) in dry, degassed DCE (1.0 ml). The reaction mixture was purged N_2 for 5 minutes and then heated at 60 °C for 5 days. The crude reaction mixture was concentrated *in vacuo*, redissolved in $CHCl_3$ (20 ml) and washed with basic aqueous EDTA (20 ml x 2), and each aqueous layer was back extracted with $CHCl_3$ (10 ml) to minimise the loss of products. The combined organic layers were washed with brine (50 ml), dried over anhydrous $MgSO_4$, filtered and concentrated *in vacuo*. The product was further purified by preparative TLC (2:90:8 CH_3OH:DCM:EtOAc *v/v*) to give *m*-**5•Zn** and *m*-**7•Zn** as dark red solids.

m-**5•Zn** Yield: 25.0 mg (30 %)
^{1}H NMR (500 MHz, $CDCl_3$, 298 K) δ: 10.23 (s, 2H, H_1), 9.33 (d, J = 4.4 Hz, 4H, H_2), 8.95 (d, J = 4.4 Hz, 4H, H_3), 8.20 (m, 2H, H_5), 7.83 (s, 2H, H_9), 7.64 (t, J = 7.6 Hz, 2H, H_6), 7.56 (s, 1H, H_4), 7.51 (s, 1H, $H_{4'}$), 7.41 (d, J = 7.6 Hz, 2H, H_7), 7.19 (m, 12H, H_{13}), 7.10 (s, 1H, H_a), 7.04 (m, 12H, H_{14}), 6.97 (m, 4H, H_{11}), 6.60 (dd, J = 15.4, 7.7 Hz, 4H, H_{12}), 6.51 (m, 6H, $H_{b,e}$), 6.21 (dd, J = 12.9, 8.2 Hz, 4H, H_f), 5.47 (s, 4H, H_{10}), 4.65 (s, 2H, H_8), 4.54 (s, 2H, $H_{8'}$), 3.89 (d, J = 16.8 Hz, 4H, $H_{c,c'}$), 3.77 (d, J = 14.8 Hz, 4H, $H_{d,d'}$), 3.48 (dt, J = 10.0, 4.8 Hz, 4H, H_g), 3.30 (app. q, J = 4.8 Hz, 4H, H_h), 3.12 (app. q, J = 4.7 Hz, 4H, H_i), 3.01 (t, J = 4.7 Hz, 4H, H_j), 2.95 (s, 4H, H_k), 1.27 (d, J = 2.6 Hz, 54H, H_{15}) ppm.
^{13}C NMR (126 MHz, $CDCl_3$, 298 K) δ: 157.9, 157.9, 157.1, 155.9, 155.8, 149.8, 149.5, 148.3, 144.2, 143.7, 143.5, 139.8, 136.7, 134.1, 132.2, 131.9, 130.7, 129.4, 129.4, 129.0, 128.9, 127.1, 126.6, 124.1, 119.7, 114.0, 113.1, 106.2, 71.6, 71.5, 70.8, 70.3, 70.2, 69.2, 66.7, 63.1, 60.9, 60.7, 34.3, 31.4, 29.7 ppm.
HR ESI-MS m/z calc'd for $[C_{145}H_{153}N_{11}O_{10}Zn]^+$, $[M+H]^+$, 2274.1199, found: 2274.1145.

m-**7•Zn** Yield: 8.0 mg (8 %)
^{1}H NMR (500 MHz, $CDCl_3$, 298 K) δ: 10.26 (d, J = 6.0 Hz, 2H, H_1), 9.35 (dd, J = 4.5, 2.1 Hz, 4H, H_2), 8.98 (t, J = 3.8 Hz, 4H, H_3), 8.17 (d, J = 7.5 Hz, 1H, H_5), 8.13 (d, J = 7.5 Hz, 1H, $H_{5'}$), 7.78 (s, 1H, H_9), 7.67 (s, 1H, $H_{9'}$), 7.62 – 7.55 (m, 3H, $H_{6,6',7}$), 7.53 (t, J = 7.7 Hz, 1H, H_a), 7.34 (d, J = 7.9 Hz, 1H, $H_{7'}$), 7.28 (t, J = 7.8 Hz, 1H, $H_{a'}$), 7.17 (dd, J = 20.5, 8.6 Hz, 14H, $H_{4,4',13,13'}$), 7.02 (dd, J = 21.1, 8.6 Hz, 14H, $H_{b,14,14'}$), 6.94 (d, J = 8.9 Hz, 2H, H_{12}), 6.88 (d, J = 8.8 Hz, 2H, $H_{12'}$), 6.82 (d, J = 8.6 Hz, 2H, $H_{b'}$), 6.71 (d, J = 11.1 Hz, 4H, H_e), 6.57 (dd, J = 11.1, 8.9 Hz, 6H, $H_{e',11}$), 6.46 (d, J = 8.9 Hz, 2H, $H_{11'}$), 6.36 (d, J = 8.2 Hz, 4H, H_f), 6.26 (d, J = 8.2 Hz, 4H, $H_{f'}$), 5.39 (d, J = 8.5 Hz, 4H, H_{10}), 4.56 (dd, J = 68.4, 34.8 Hz, 4H, $H_{8,8'}$), 4.23 – 3.78 (m, 16H, $H_{c,d}$), 3.69 – 3.04 (m, 40H, $H_{g\text{-}h}$), 1.26 (d, J = 10.5 Hz, 54H, H_{15}) ppm.
^{13}C NMR (126 MHz, $CDCl_3$, 298 K) δ: 158.4, 158.1, 158.0, 157.7, 157.4, 157.2, 156.1, 156.0, 149.9, 149.8, 149.5, 148.2, 148.2, 144.2, 144.2, 143.9, 143.1, 143.0, 139.5, 139.4, 136.9, 136.7, 134.1, 133.9, 133.8, 133.8, 132.6, 132.0, 131.9, 131.8, 130.7, 130.6, 129.9, 129.6, 129.5, 129.2, 129.1, 127.2, 127.1, 126.8, 124.2, 124.0, 124.0, 120.5, 120.0, 119.8, 119.2, 119.1, 114.5, 114.2, 114.1, 113.2, 113.1, 106.2, 72.0, 71.9, 71.7, 71.2, 71.0, 70.9, 70.7, 70.7, 70.3, 70.2, 69.6, 69.3, 69.2, 67.5, 66.9, 66.8, 63.0, 62.9, 61.3, 61.3, 34.3, 31.4 ppm.
HR ESI-MS m/z calc'd for $[C_{176}H_{192}N_{12}O_{18}Zn]^+$, $[M + H]^+$, 2827.3875, found: 2827.3808.

Para-substituted Zn(II) Metalloporphyrin [2]-and [3]-Rotaxanes *p*-**6•Zn**, *p*-**8•Zn**

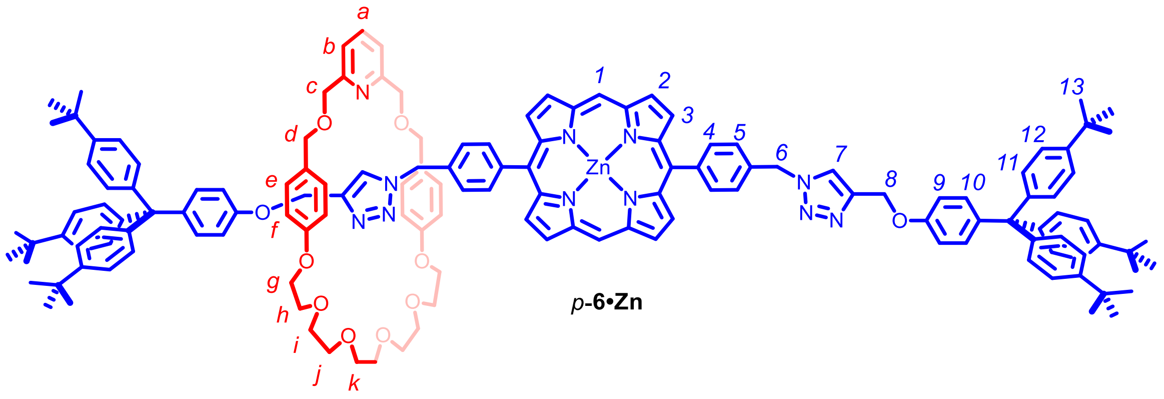

p-**6•Zn**

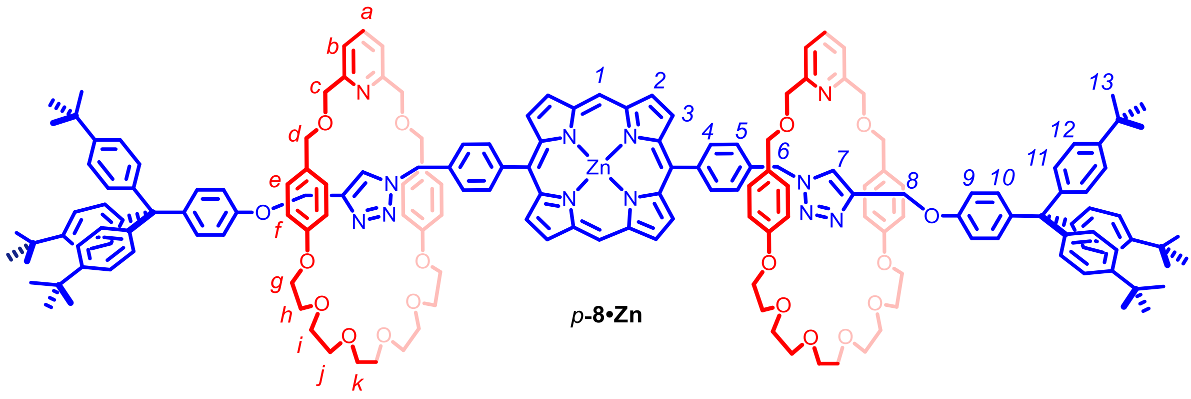

p-**8•Zn**

Macrocycle **2** (10.0 mg, 0.018 mmol) and [Cu(MeCN$_4$)]PF$_6$ (6.7 mg, 0.0196 mmol) were added to dry, degassed DCE (1.6 mL) in a sealed flask. The mixture was purged with N$_2$ for 10 min, and stirred at room temperature for 30 min. Stopper alkyne[3] (59.0 mg, 0.108 mmol) and the metalloporphyrin bis-azide *p*-**4•Zn** (34.0 mg, 0.054 mmol) were dissolved in degassed DCE (3 mL) and the resulting solution purged with N$_2$ for 5 min. The porphyrin solution was added dropwise to the copper solution, and the reaction mixture heated to 60 °C for 3 days. The reaction was cooled to room temperature and the solvent removed *in vacuo*. The residue was taken up in DCM (30 mL) and washed with basic aqueous EDTA (2 x 20 mL) and water (20 mL). The combined aqueous layers were re-extracted with further DCM (20 mL) and the combined organic layers dried over MgSO$_4$ and the volatiles removed *in vacuo*. The products were obtained by preparative TLC (4% MeOH *v/v* in DCM, followed by 2:89:9 MeOH:DCM:EtOAc *v/v*) as red solids.

p-**6•Zn** Yield: 31.2 mg (76%)
^{1}H NMR (500 MHz, CDCl$_3$, 298 K) δ: 10.31 (s, 2H, H_1), 9.36 (d, J = 4.4 Hz, 4H, H_2), 8.89 (d, J = 4.4 Hz, 4H, H_3), 7.98 (d, J = 7.5 Hz, 4H, H_4), 7.28 (m, 15H, $H_{11,a,b}$), 7.11 (m, 20H, $H_{5,9,12}$), 6.99 (m, 8H, $H_{10,e}$), 6.57 (d, J = 8.2 Hz, 4H, H_f), 6.49 (broad s, 4H, H_8), 5.05 (broad s, 4H, H_6), 4.43 (s, 4H, H_c), 4.25 (s, 4H, H_d), 3.89 (t, J = 5.0 Hz, 4H, H_g), 3.71 (t, J = 5.0 Hz, 4H, H_h), 3.59 (t, J = 5.0 Hz, 4H, H_i), 3.55 (t, J = 5.0 Hz, 4H, H_j), 3.53 (s, 4H, H_k), 1.33 (s, 54H, H_{13}) ppm. **^{13}C{^{1}H} NMR** (126 MHz, CDCl$_3$, 298 K) δ: 158.3, 157.6, 149.7, 149.6, 148.4, 144.2, 141.6,

139.9, 139.3, 136.9, 134.9, 132.2, 131.7, 130.9, 130.8, 130.7, 129.8, 129.5, 128.8, 124.1, 124.1, 120.0, 115.3, 114.5, 114.4, 114.1, 113.2, 113.0, 106.2, 72.2, 71.3, 70.8, 70.7, 70.7, 69.5, 67.2, 63.1, 34.4, 31.4 ppm.
HR ESI-MS m/z calc'd for $[C_{145}H_{154}N_{11}O_{10}Zn]^+$, $[M+H]^+$, 2274.1200 found: 2274.1237.

p-**8•Zn** Yield: 13.2 mg (24%)
^{1}H NMR (500 MHz, $CDCl_3$, 298 K) δ: 10.30 (s, 2H, H_1), 9.37 (d, J = 4.5 Hz, 4H, H_2), 8.95 (d, J = 4.5 Hz, 4H, H_3), 7.96 (d, J = 7.6 Hz, 4H, H_4), 7.82 (s, 2H, H_7), 7.29 (t, J = 7.8 Hz, 2H, H_a), 7.24 (d, J = 8.6 Hz, 12H, H_{11}), 7.22 (d, J = 7.6 Hz, 2H, H_5), 7.11 (d, J = 8.6 Hz, 12H, H_{12}), 7.07 (m, 8H, $H_{9,b}$), 7.03 (d, J = 8.3 Hz, 8H, H_e), 6.75 (d, J = 8.1 Hz, 4H, H_{10}), 6.61 (d, J = 8.3 Hz, 8H, H_f), 5.40 (s, 4H, H_6), 4.88 (s, 4H, H_8), 4.46 (s, 8H, H_c), 4.23 (m, 8H, H_d), 3.93 (m, 8H, H_g), 3.73 (m, 8H, H_h), 3.61 (m, 8H, H_i), 3.57 (m, 8H, H_j), 3.54 (app. s, 8H, H_k), 1.31 (s, 54H, H_{15}) ppm.
^{13}C{^{1}H} NMR (126 MHz, $CDCl_3$, 298 K) δ: 158.4, 157.6, 156.3, 149.9, 149.5, 148.3, 144.2, 144.1, 139.7, 142.3, 136.9, 134.8, 134.3, 132.8, 132.7, 132.2, 131.8, 130.9, 130.7, 129.8, 129.6, 126.3, 124.3, 124.1, 120.0, 119.4, 114.4, 113.3, 72.2, 71.3, 70.8, 70.8, 70.7, 69.6, 67.2, 63.1, 61.5, 34.3, 31.4 ppm.
HR ESI-MS Mass calc'd for $[C_{176}H_{192}N_{12}O_{18}Zn]^{2+}$, $[M+2H]^{2+}$: 1414.1974, found: 1414.1989.

<u>*Para*-substituted Free Base [2]Rotaxane *p*-**6•H₂**</u>

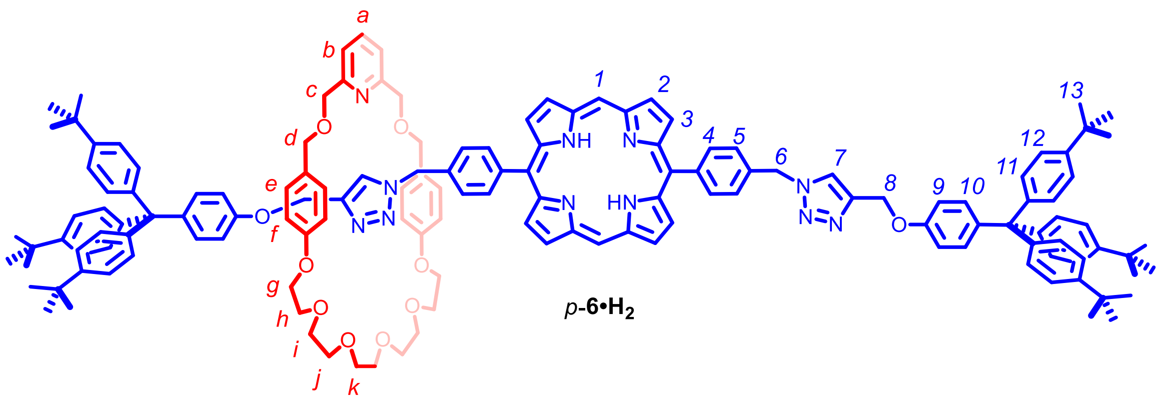

p-**6•H$_2$**

Trifluoroacetic acid (0.05 ml, 0.66 mmol) was added dropwise to a solution of *p*-**6•Zn** (15 mg, 0.0066 mmol) in dry DCM (5 ml) in a sealed flask before stirring for four hours at room temperature. Aqueous $NaHCO_3$ (1.0 M) was added to the reaction mixture until effervescence ceased, whereupon an additional 10 ml was added. The organic layer was collected and the aqueous layer further extracted with DCM (10 ml x 3). The combined organic layers were washed with 1.0 M $NaHCO_3$ (20 ml) and H_2O (30 ml x 4) and dried over MgSO4. The solvent was removed *in vacuo* and the crude product purified by preparative thin layer chromatography (2:8:90 MeOH:EtOAc:DCM *v/v*), *p*-**6•H$_2$** was afforded as a red solid. Yield: 13.3 mg (91%).

^{1}H NMR (400 MHz, $CDCl_3$, 298 K) δ: 10.31 (s, 2H, H_1), 9.37 (d, J = 4.6 Hz, 4H, H_2), 8.99 (d, J = 4.6 Hz, 4H, H_3), 8.12 (s, 4H, H_4), 7.88 (s, 2H, H_7), 7.49 (s, 4H, H_5), 7.33 (t, J = 7.7 Hz, 1H, H_a), 7.23 (d, J = 8.6 Hz, 12H, H_{11}), 7.19 – 7.07 (m, 18H, $H_{b,10,12}$), 7.06 (d, J = 8.3 Hz, 4H, H_9),

6.88 (d, J = 8.6 Hz, 4H, H_e), 6.65 – 6.58 (d, J = 8.6 Hz, 4H, H_f), 5.67 (s, 2H, H_6), 5.16 (s, 2H, H_8), 4.51 (s, 4H, H_c), 4.35 (s, 4H. H_d), 3.93 (t, J = 4.8 Hz, 4H, H_g), 3.73 (t, J = 4.8 Hz, 4H, H_h), 3.65 – 3.55 (m, 12H, $H_{i,\,j,\,k}$), 1.30 (s, 54H, H_{13}), -3.16 (s, 2H, NH) ppm.

^{13}C{^{1}H} NMR (101 MHz, $CDCl_3$, 298 K) δ 158.4, 158.3 157.6, 156.3, 156.3 148.3, 147.0, 144.1, 140.0, 135.2, 134.4, 132.3, 132.3, 131.8, 131.2, 130.8, 130.0, 129.9, 129.6, 126.7, 124.1, 120.1, 114.4, 113.3, 113.3, 105.4, 72.3, 71.5, 70.8, 70.7, 69.6, 67.2, 63.1, 61.9, 53.8, 34.3, 31.4, 29.7 ppm.
HR ESI-MS m/z calc'd for $[C_{145}H_{156}N_{10}O_{10}]^+$, $[M+H]^+$: 2211.2064, found 2211.2051.

<u>*Para*-substituted Ni(II) Metalloporphyrin [2]Rotaxane *p*-**6•Ni**</u>

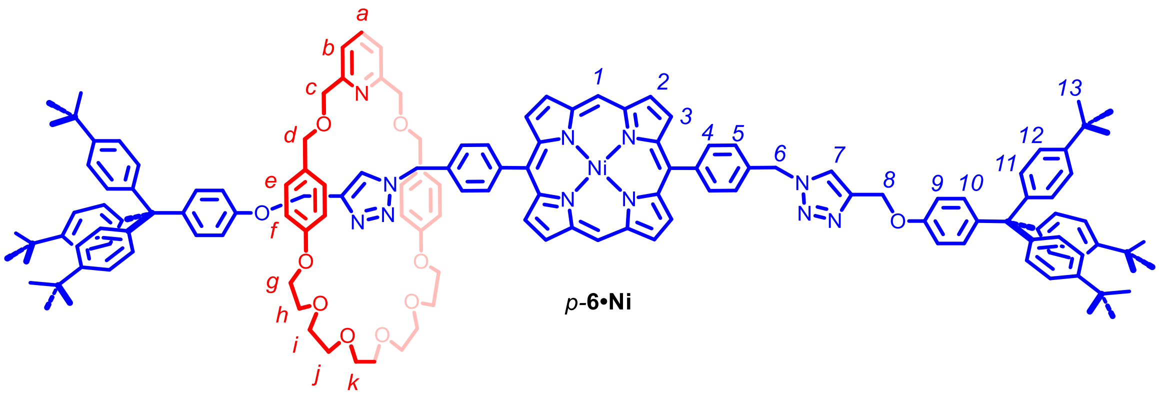

p-**6•H₂** (11.8 mg, 0.005 mmol) and $Ni(acac)_2$ (30.0 mg, 0.16 mmol) were dissolved in dry degassed DMF (1.5 ml) in a sealed flask before heating at reflux for four hours. The reaction mixture cooled to room temperature and the volatiles removed *in vacuo*. The red residual solid was taken up in DCM (20 ml) and washed with H_2O (50 ml). The aqueous layer was back extracted with DCM (20 ml x 3) to minimise loss of product. The combined organic layer was washed with H_2O (40 ml x 4), dried over $MgSO_4$. The volatiles were removed *in vacuo* and the product obtained by purifying by preparative thin layer chromatography (2:8:90 MeOH:EtOAc:DCM *v/v*). Yield: 10.5 mg (87%).

^{1}H NMR (600 MHz, $CDCl_3$, 298 K) δ: 9.93 (s, 2H, H_1), 9.16 (d, J = 4.7 Hz, 4H, H_2), 8.82 (d, J = 4.7 Hz, 4H, H_3), 7.91 (d, J = 7.3 Hz, 4H, H_4), 7.84 (s, 2H, H_7), 7.39 (d, J = 7.3 Hz, 4H, H_5), 7.29 (t, J = 7.8 Hz, 1H, H_a), 7.24-7.21 (m, 14H, $H_{11,\,b}$), 7.15-7.08 (d, 16H, $H_{10,12}$), 7.04 (d, J = 8.6 Hz, 4H, H_9), 6.86 (d, J = 8.8 Hz, 4H, H_e), 6.59 (d, J = 8.8 Hz, 4H, H_f), 5.61 (s, 4H, H_6), 5.14 (s, 4H, H_8), 4.49 (s, 4H, H_c), 4.30 (s, 4H, H_d), 3.91 (t, J = 4.8 Hz, 4H, H_g), 3.72 (t, J = 4.8 Hz, 4H, H_h), 3.67 – 3.53 (m, 12H, $H_{i,\,j,\,k}$), 1.30 (s, 54H, H_{13}) ppm.
^{13}C{^{1}H} NMR (151 MHz, $CDCl_3$, 298 K) δ: 158.4, 157.6, 156.3, 148.4, 144.6, 144.2, 142.7, 142.6, 141.2, 140.1, 137.0, 134.4, 134.2, 132.5, 132.3, 132.2, 130.7, 129.9, 129.6, 126.6, 124.1, 123.6, 120.1, 117.5, 114.4, 113.3, 105.2, 72.3, 71.5, 70.8, 70.7, 70.7, 69.5, 67.2, 63.1, 61.9, 53.8, 50.9, 34.3, 31.4 ppm.
HR ESI-MS m/z calc'd for $[C_{145}H_{154}N_{11}O_{10}Ni]^+$, $[M+H]^+$: 2267.1228, found: 2267.1250.

__*Meta*-substituted Zn(II) Metalloporphyrin Axle *m*-9•Zn__

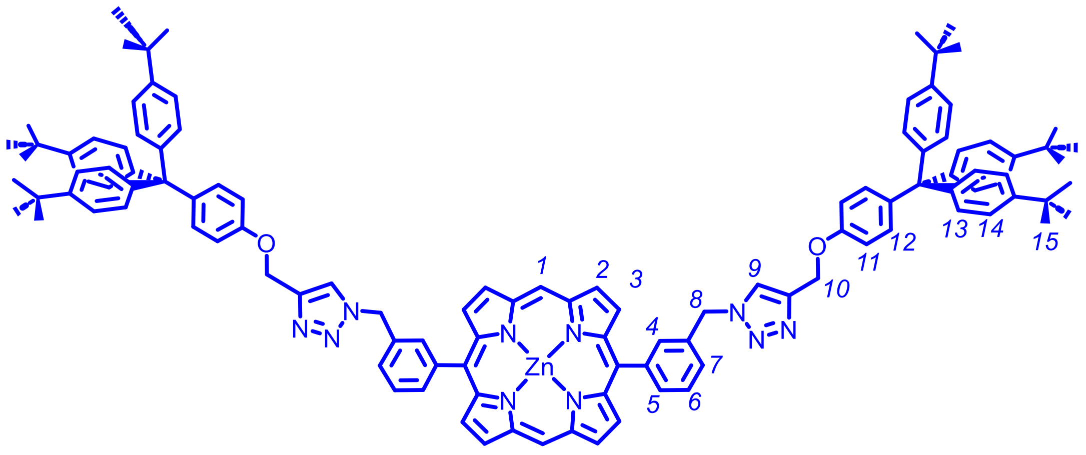

m-**9•Zn**

Bis-azido zinc(II) metalloporphyrin *m*-**3•Zn** (100 mg, 0.157 mmol) was dissolved in dry THF (3 ml) in a sealed flask and degassed for five minutes. TBTA (50 mg, 0.095 mmol, 0.6 equiv.) and [Cu(MeCN$_4$)]PF$_6$ (46.9 mg, 0.126 mmol) were added and the solution was degassed for a further five minutes. A solution of stopper-alkyne[3] (179 mg, 0.330 mmol) in dry and degassed THF (2.0 ml) was prepared and added into the microwave vial before degassing for five minutes. The solution was stirred under positive pressure of N_2 for three days at room temperature. The reaction mixture was diluted with DCM (40 ml), washed with basic aqueous EDTA (30ml x 2), H_2O (30 ml) and brine (30 ml), then dried over anhydrous $MgSO_4$ and filtered. The volatiles was removed *in vacuo* and the product purified by column chromatography (3:7 n-hexanes:DCM *v/v* graded to 100 % DCM and then to 1:9 EtOAc:DCM). Yield: 0.116 g (43 %).

^{1}H NMR (500 MHz, CDCl$_3$, 298 K) δ: 9.96 (d, J = 5.4 Hz, 2H, H_1), 9.05 (d, J = 4.5 Hz, 4H, H_2), 8.66 (d, J = 4.5 Hz, 4H, H_3), 8.05 (t, J = 8.7 Hz, 2H, H_5), 7.62 (s, 2H, H_9), 7.44 (m, 2H, H_6), 7.29 – 7.21 (m, 12H, H_{13}), 7.14 – 7.06 (m, 12H, H_{14}), 7.05 (d, J = 8.8 Hz, 4H, H_{12}), 7.00 (d, J = 6.8 Hz, 2H, H_7), 6.86 (s, 2H, H_4), 6.32 (dd, J = 8.8, 4.1 Hz, 4H, H_{11}), 4.99 – 4.82 (m, 4H, H_{10}), 3.39 (s, 4H, H_8), 1.31 (d, J = 2.1 Hz, 54H, H_{15}) ppm. **^{13}C{^{1}H} NMR** (126 MHz, CDCl$_3$, 298 K) δ: 171.2, 155.6, 149.8, 149.5, 148.5, 144.2, 144.1, 143.1, 140.1, 135.0, 133.9, 132.3, 131.9, 130.9, 127.0, 126.3, 124.3, 121.6, 118.4, 113.1, 106.2, 63.2, 60.5, 60.0, 53.9, 34.5, 31.6 ppm.
HR ESI-MS m/z calc'd for [$C_{114}H_{114}N_{10}NaO_2Zn$]$^+$, [M+Na]$^+$: 1742.8344, found: 1742.8282.

Para-substituted Zn(II) Metalloporphyrin Axle *p*-**10•Zn**

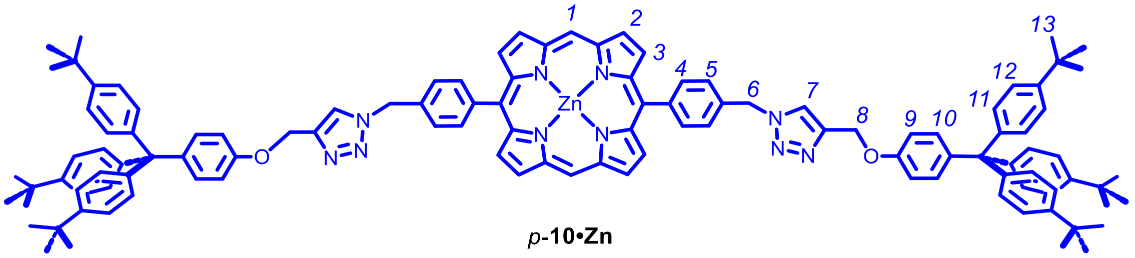

p-**10•Zn**

Bis-azido zinc(II) metalloporphyrin *p*-**4•Zn** (200 mg, 0.314 mmol) was dissolved in dry and degassed THF (8 ml) in a sealed flask and degassed for five minutes. TBTA (100 mg, 0.189 mmol) and $[Cu(MeCN_4)]PF_6$ (93.7 mg, 0.252 mmol) were added and the solution was degassed for a further five minutes. A solution of stopper-alkyne[3] (358 mg, 0.660 mmol) in dry and degassed THF (4.0 ml) was prepared and added into the microwave vial before degassing for five minutes. The solution was stirred under positive pressure of N_2 for two days at room temperature. The reaction mixture was diluted with DCM (40 ml), washed with basic aqueous EDTA (30ml x 2), brine (30 ml) and H_2O (30 ml). The combined organic fractions were dried over $MgSO_4$ and the volatiles removed *in vacuo*. The product was purified by column chromatography (3:7 hexanes:DCM *v/v* graded to 1:9 EtOAc:DCM *v/v*) to obtain the product as a purple semi-crystalline solid. Yield 0.252 g (47%)

^{1}H NMR (400 MHz, $CDCl_3$, 298 K) δ: 10.23 (s, 2H, H_1), 9.38 (d, J = 4.4 Hz, 4H, H_2), 9.02 (d, J = 4.4 Hz, 4H, H_3), 8.23 (d, J = 6.5 Hz, 4H, H_4), 7.75 (s, 2H, H_7), 7.57 (d, J = 6.5 Hz, 4H, H_5), 7.27 (d, J = 7.9 Hz, 12H, H_{11}), 7.20 (d, J = 8.4 Hz, H_{10}), 7.16 (t, J = 7.9 Hz, 12H, H_{12}), 6.93 (d, J = 8.4 Hz, 4H, H_9), 5.80 (s, 4H, H_6), 5.20 (s, 4H, H_8), 1.32 (s, 54H, H_{13}) ppm.
^{13}C{^{1}H} NMR (101 MHz, $CDCl_3$, 298 K) δ: 156.3, 149.9, 149.6, 148.5, 145.0, 144.2, 144.0, 140.4, 135.5, 133.5, 132.5, 132.1, 131.8, 130.9, 126.2, 124.2, 123.0, 118.5, 113.4, 106.1, 63.2, 62.2, 54.3, 34.4, 31.5 ppm.
HR ESI-MS: m/z calculated for $[C_{114}H_{114}N_{10}NaO_2Zn]^+$, $[M+Na]^+$: 1741.8315, found: 1741.8329.

S3 Spectral Characterisation of Novel Structures

2

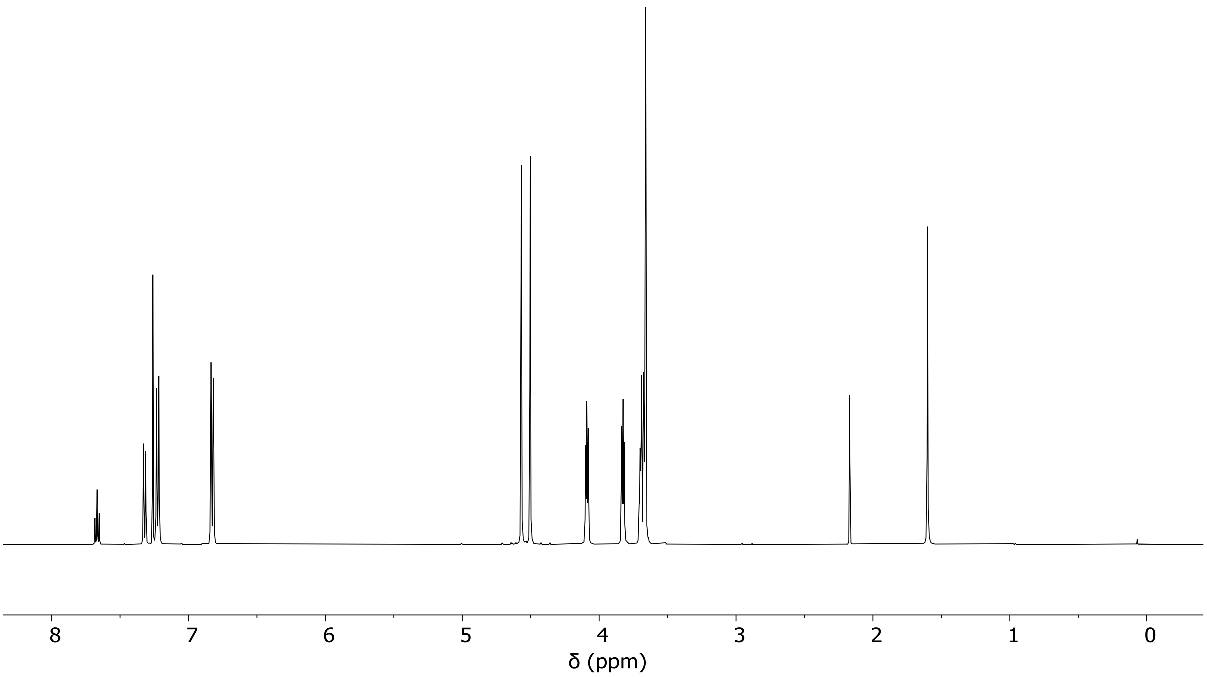

Figure S1. ^{1}H NMR spectrum (400 MHz, $CDCl_3$, 298 K) of **2**.

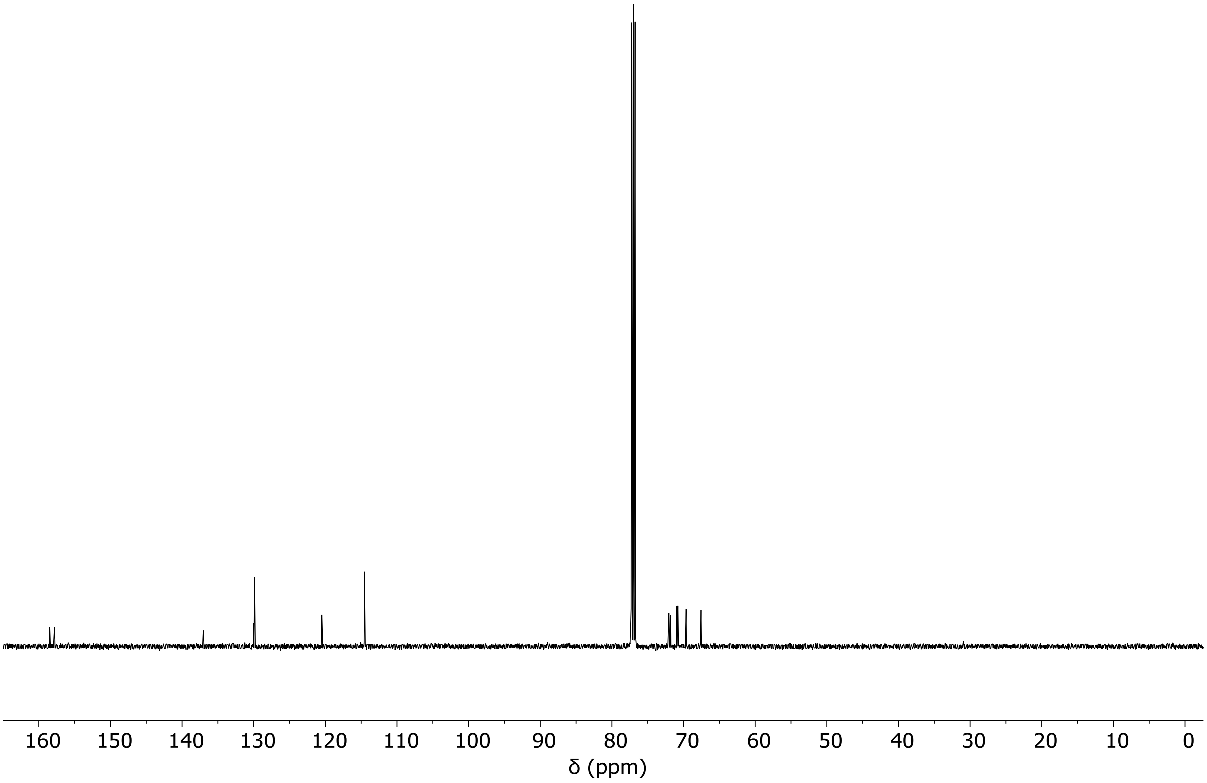

Figure S2. ^{13}C{^{1}H} NMR spectrum (126 MHz, $CDCl_3$, 298 K) of **2**.

m-**3•H2**

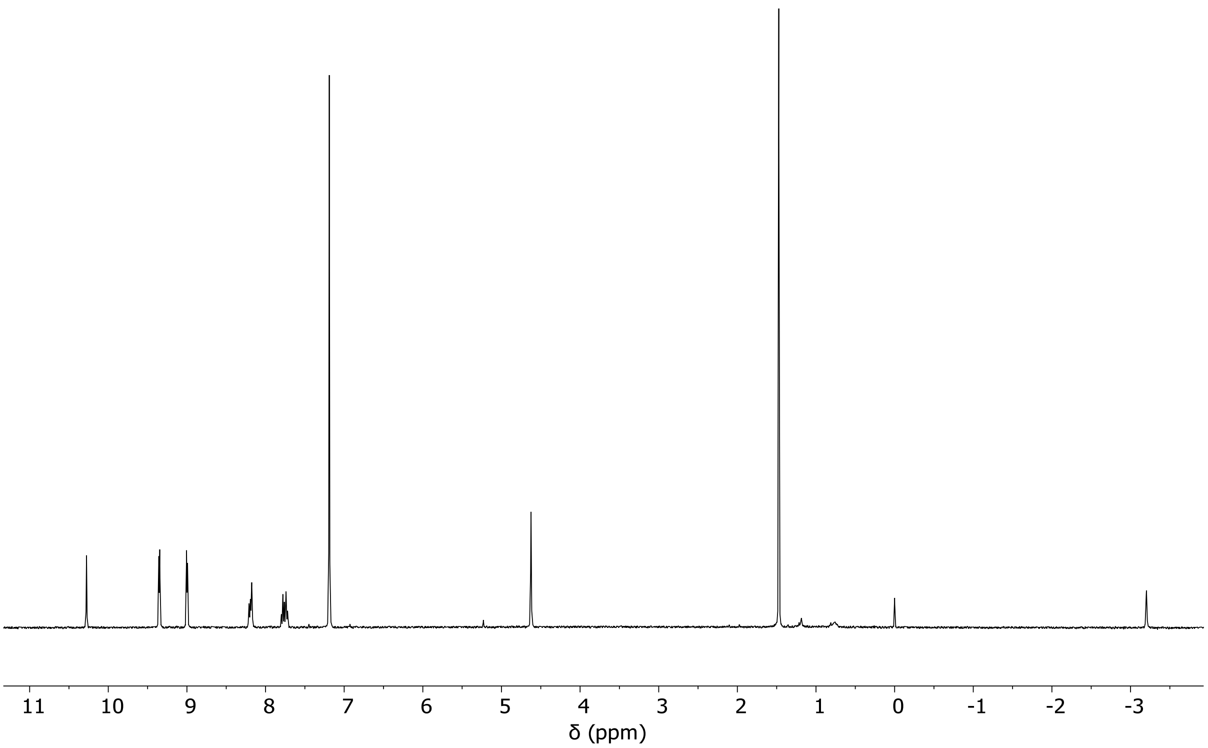

Figure S3. ^{1}H NMR spectrum (400 MHz, $CDCl_3$, 298 K) of *m*-**3•H2**.

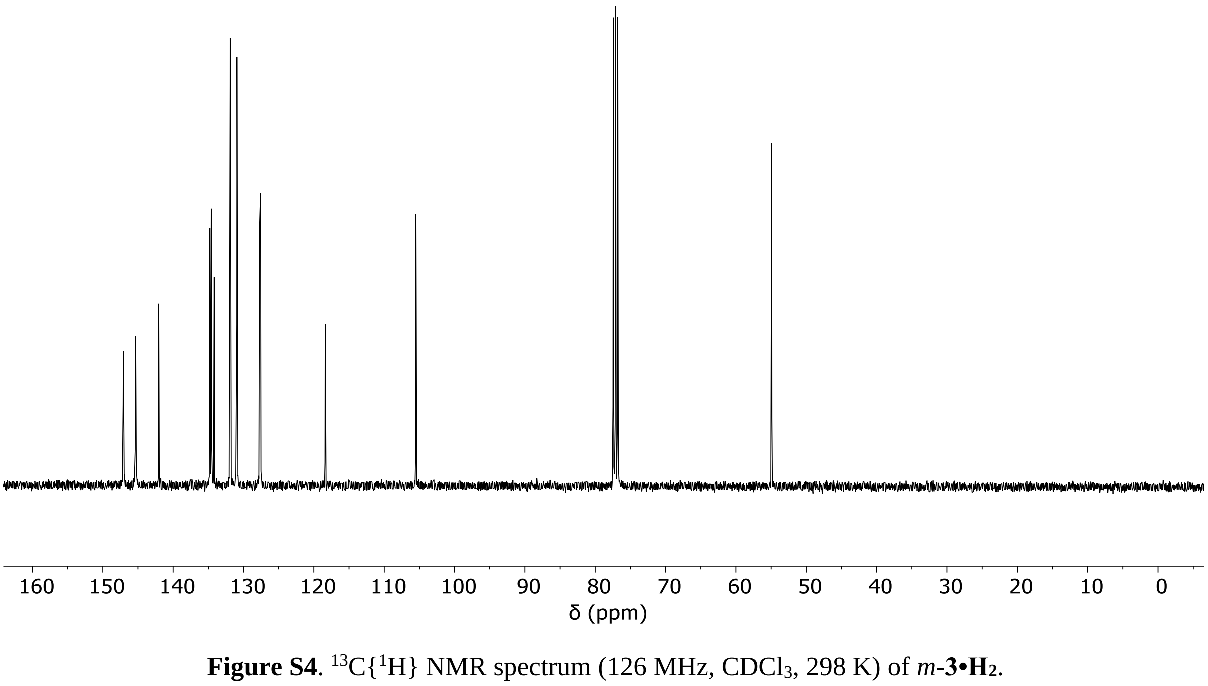

Figure S4. ^{13}C{^{1}H} NMR spectrum (126 MHz, $CDCl_3$, 298 K) of *m*-**3•H2**.

m-**3•Zn**

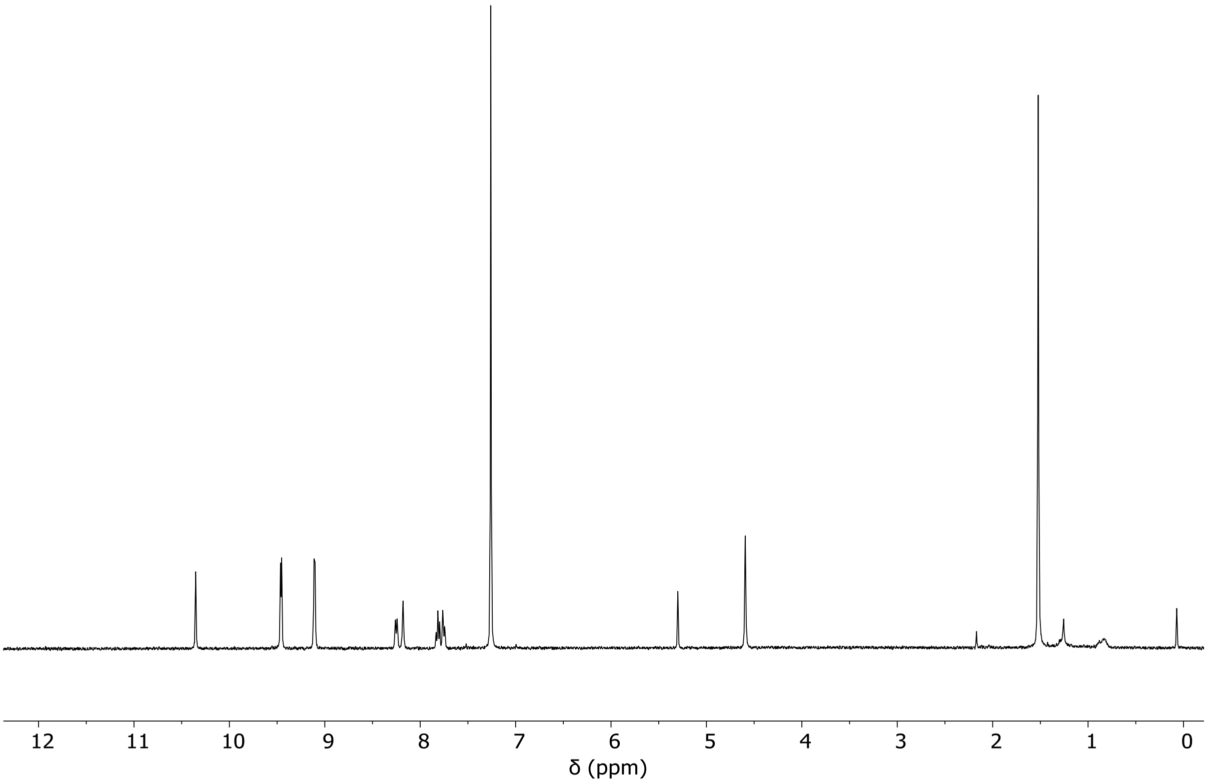

Figure S5. ^{1}H NMR spectrum (400 MHz, $CDCl_3$, 298 K) of *m*-**3•Zn**.

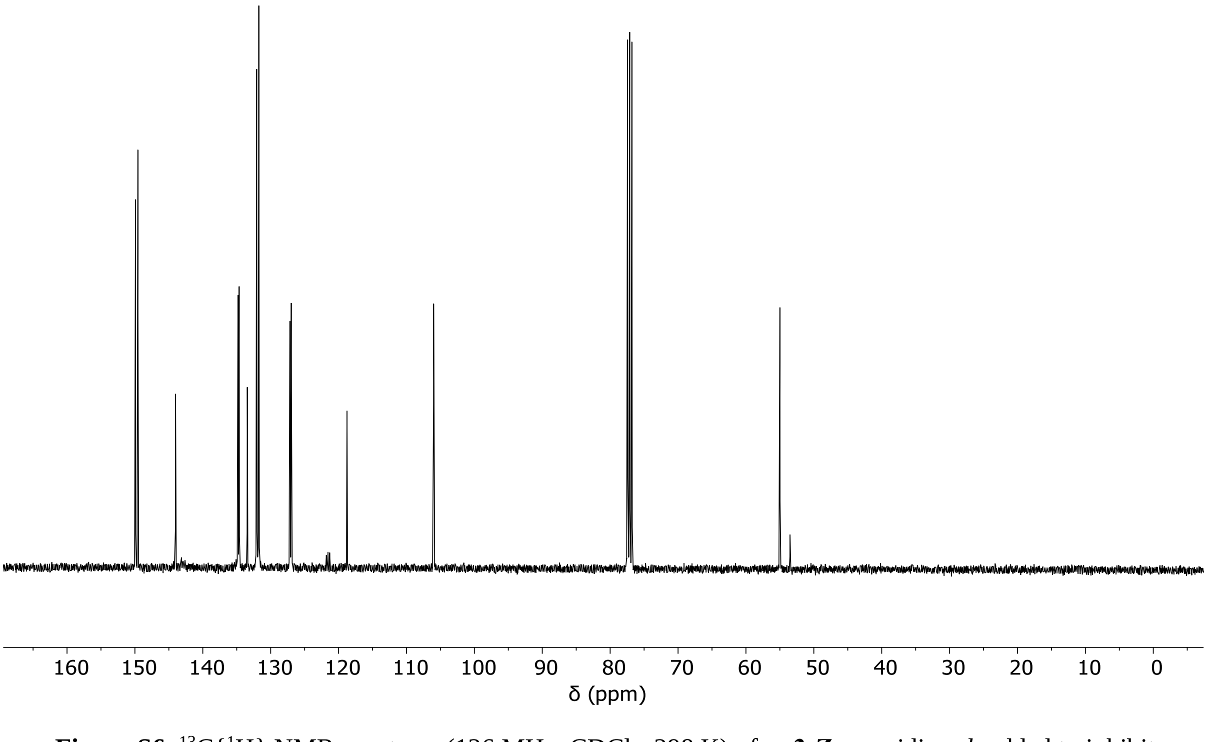

Figure S6. $^{13}C\{^{1}H\}$ NMR spectrum (126 MHz, $CDCl_3$, 298 K) of *m*-**3•Zn**, pyridine-d_5 added to inhibit aggregation.

m-**5•Zn**

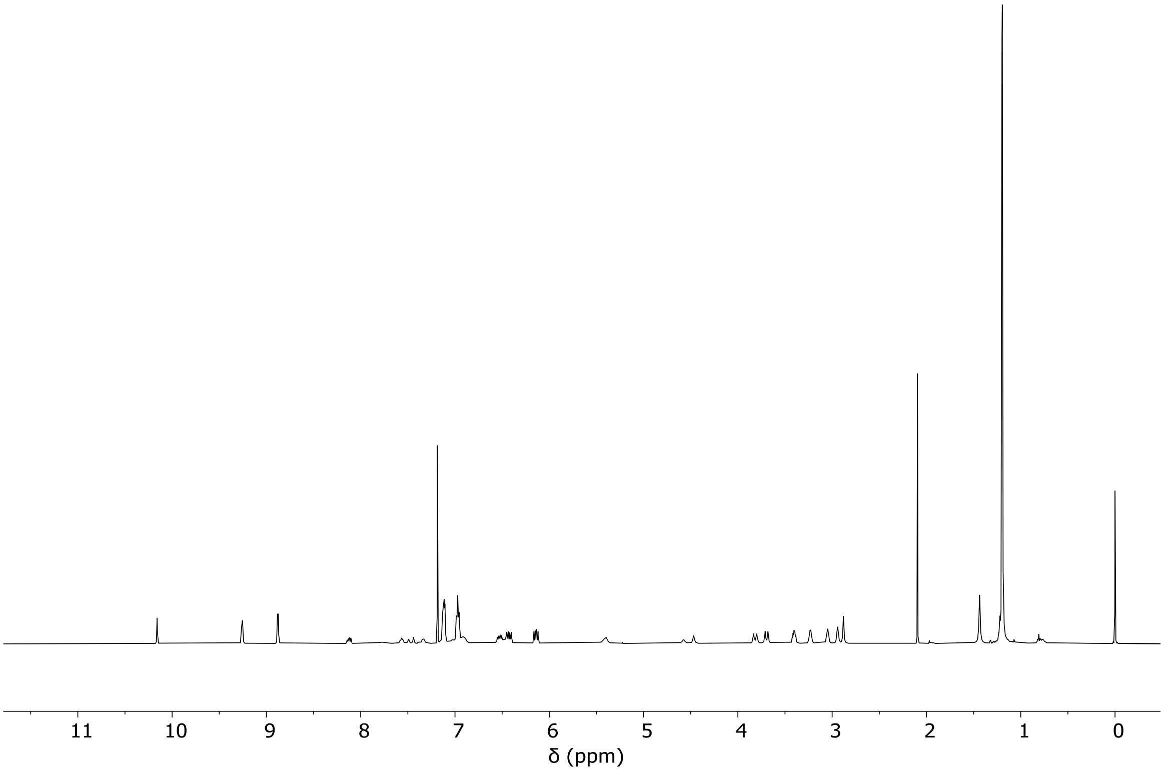

Figure S7. ^{1}H NMR spectrum (400 MHz, $CDCl_3$, 298 K) of *m*-**5•Zn**.

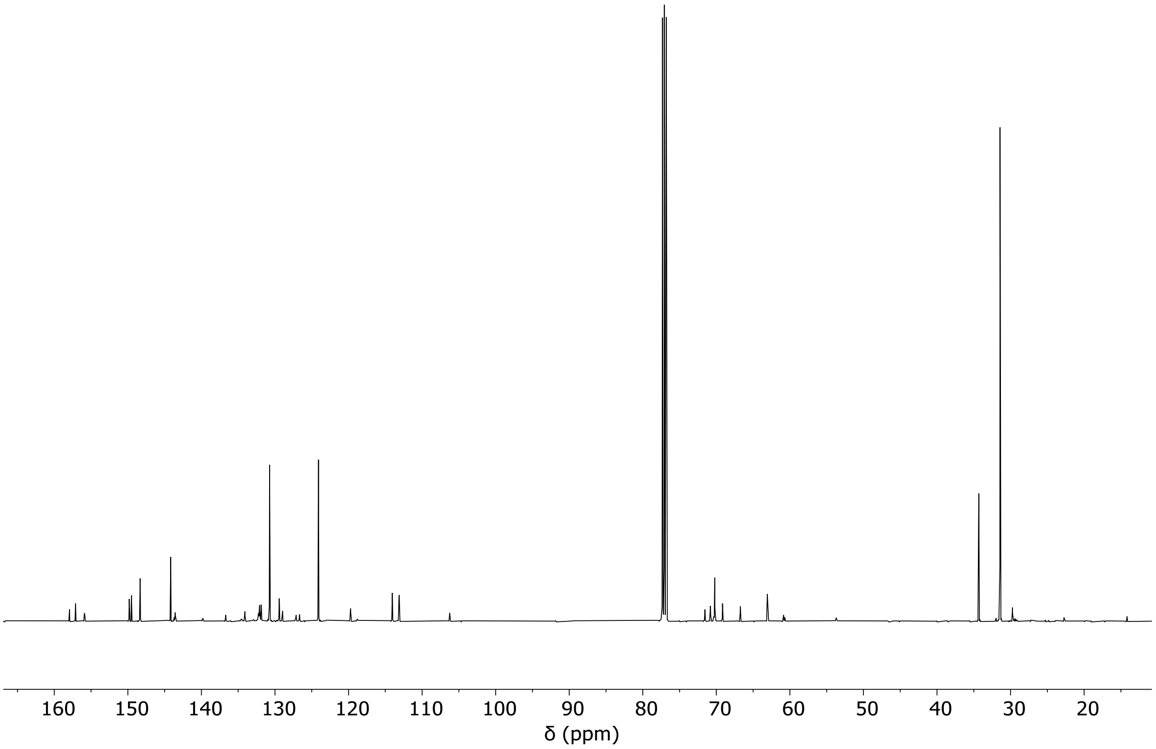

Figure S8. $^{13}C\{^{1}H\}$ NMR spectrum (126 MHz, $CDCl_3$, 298 K) of *m*-**5•Zn**.

p-**6•Zn**

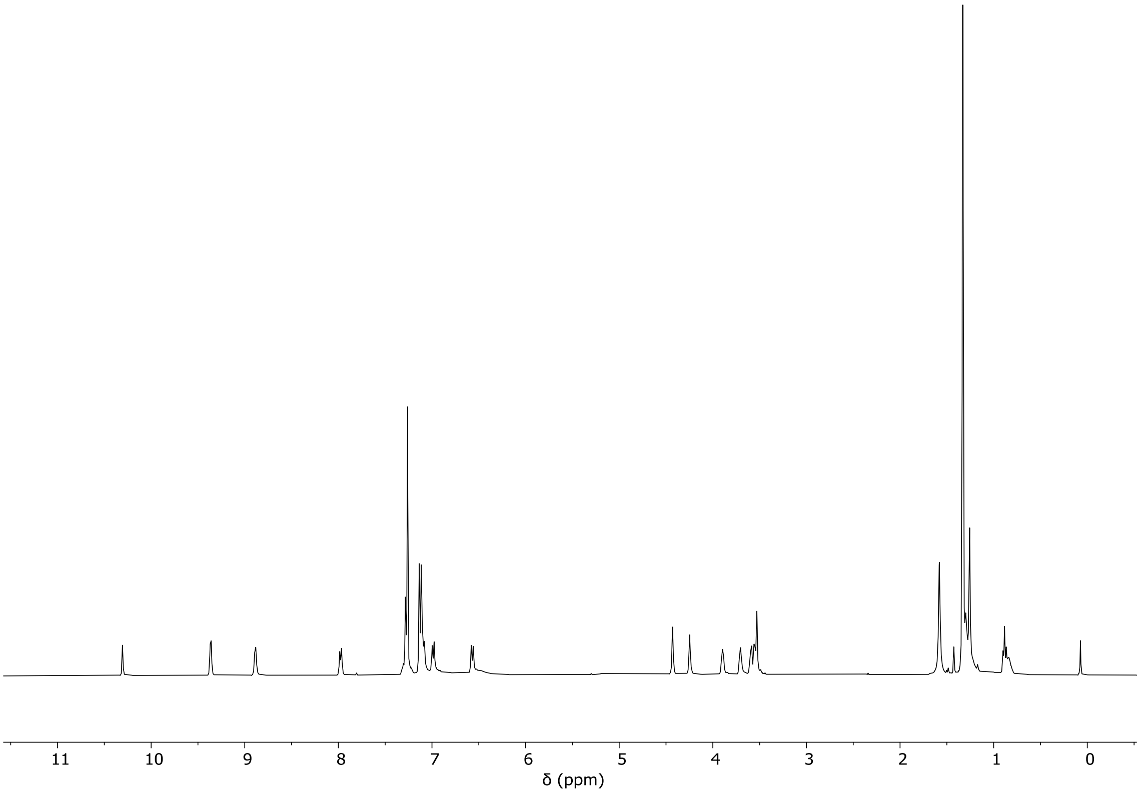

Figure S9. ^{1}H NMR spectrum (400 MHz, $CDCl_3$, 298 K) of *p*-**6•Zn**.

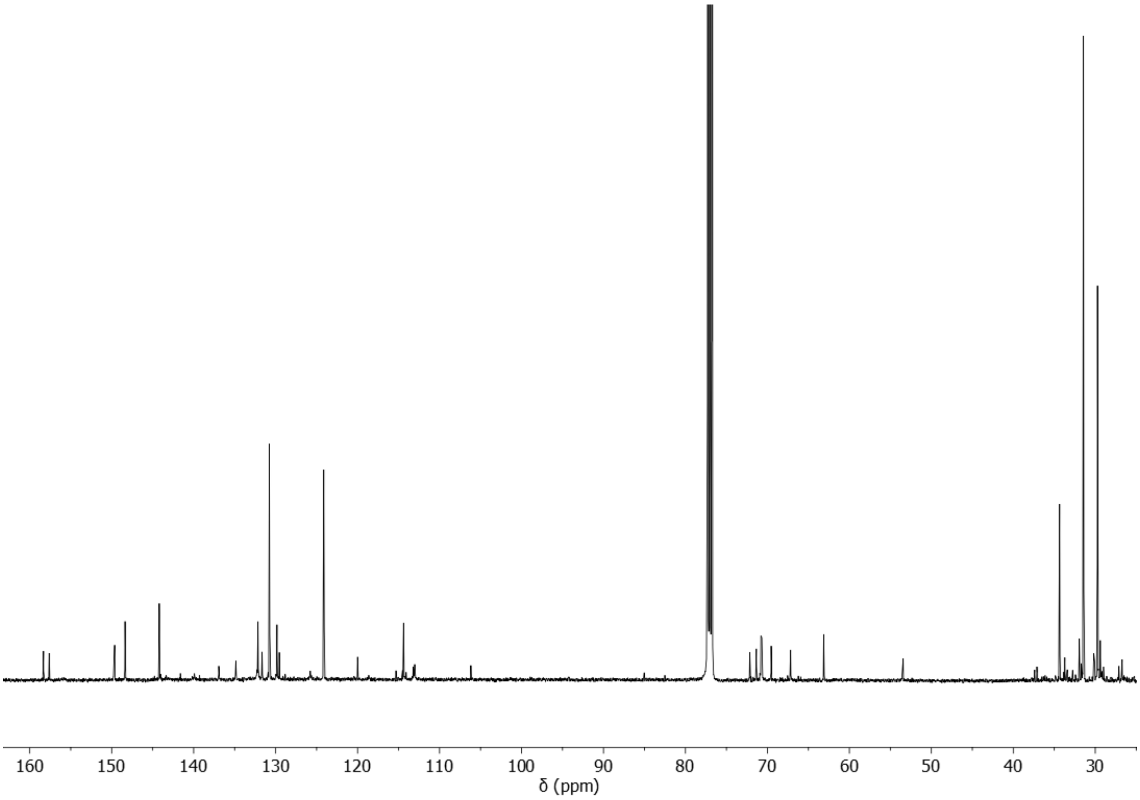

Figure S10. ^{13}C{^{1}H} NMR spectrum (126 MHz, $CDCl_3$, 298 K) of *p*-**6•Zn**.

m-**7•Zn**

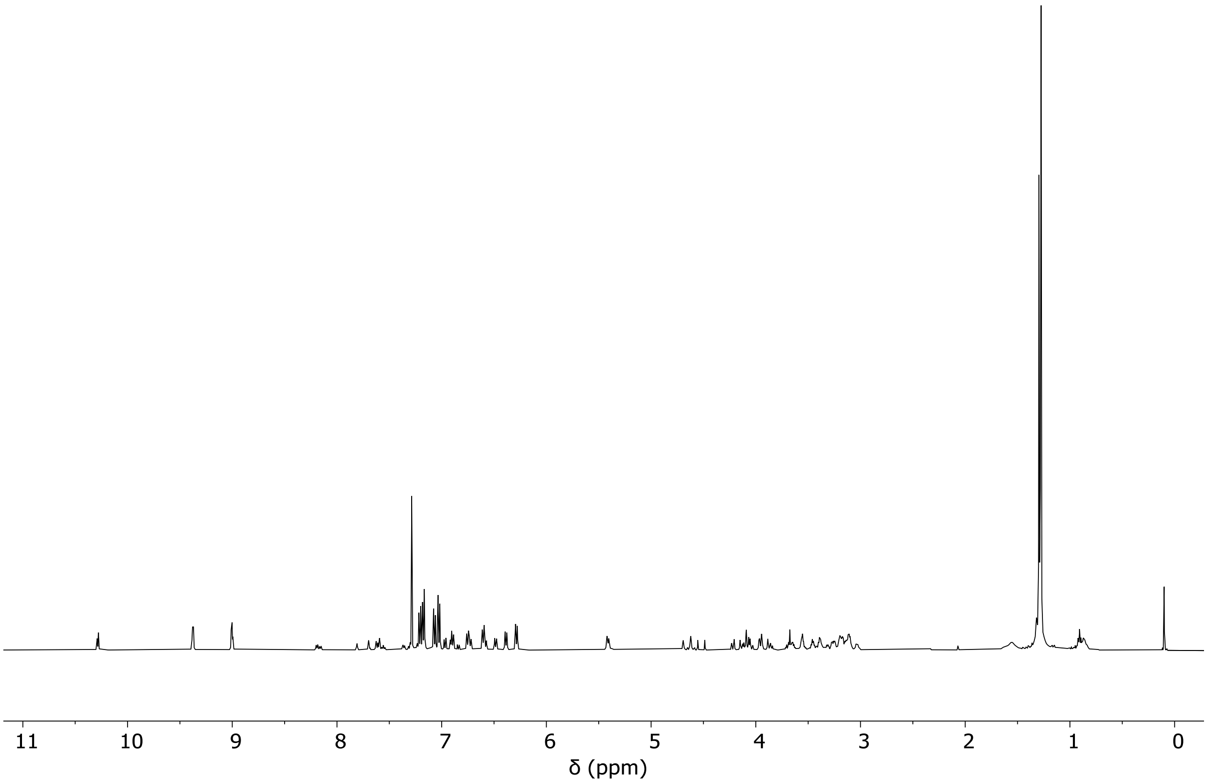

Figure S11. ^{1}H spectrum (500 MHz, $CDCl_3$, 298 K) of *m*-**7•Zn**.

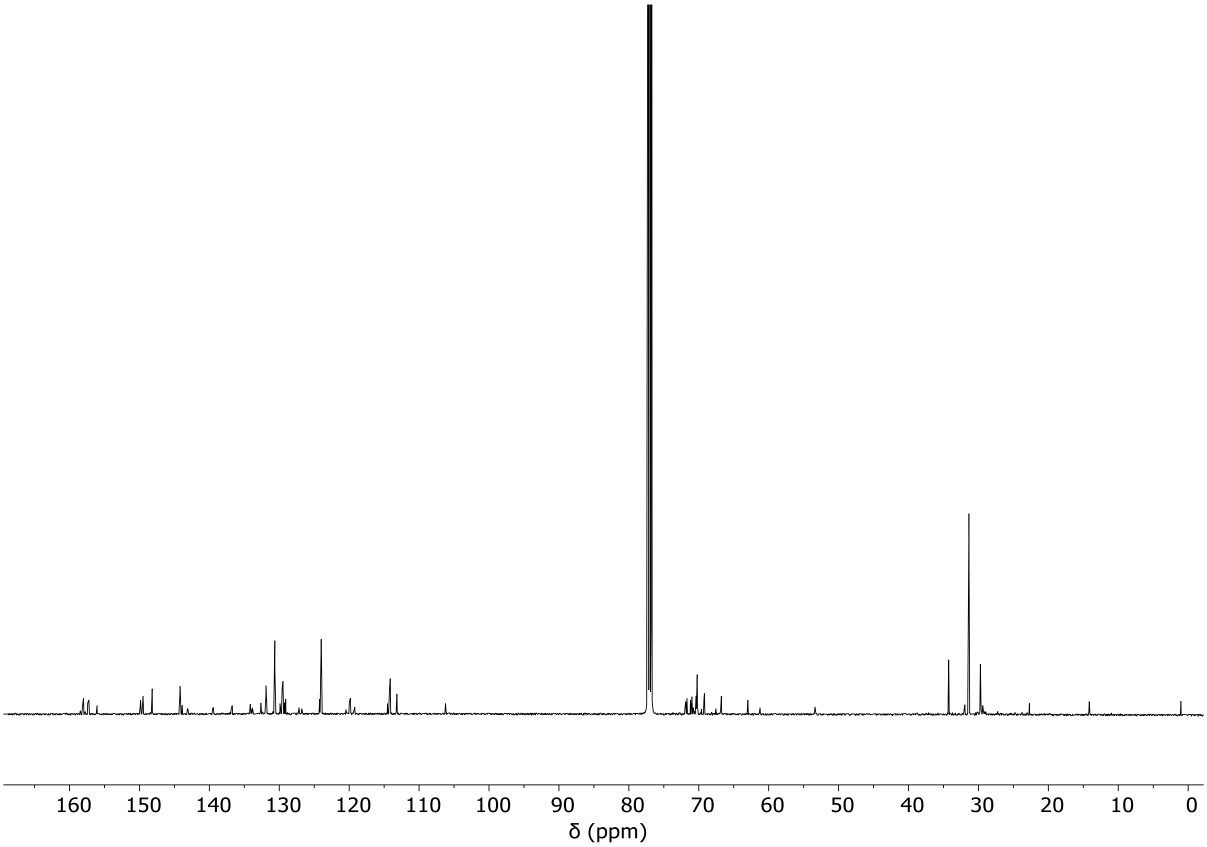

Figure S12. $^{13}C\{^{1}H\}$ NMR spectrum (126 MHz, $CDCl_3$, 298 K) of *m*-**7•Zn**.

p-**8•Zn**

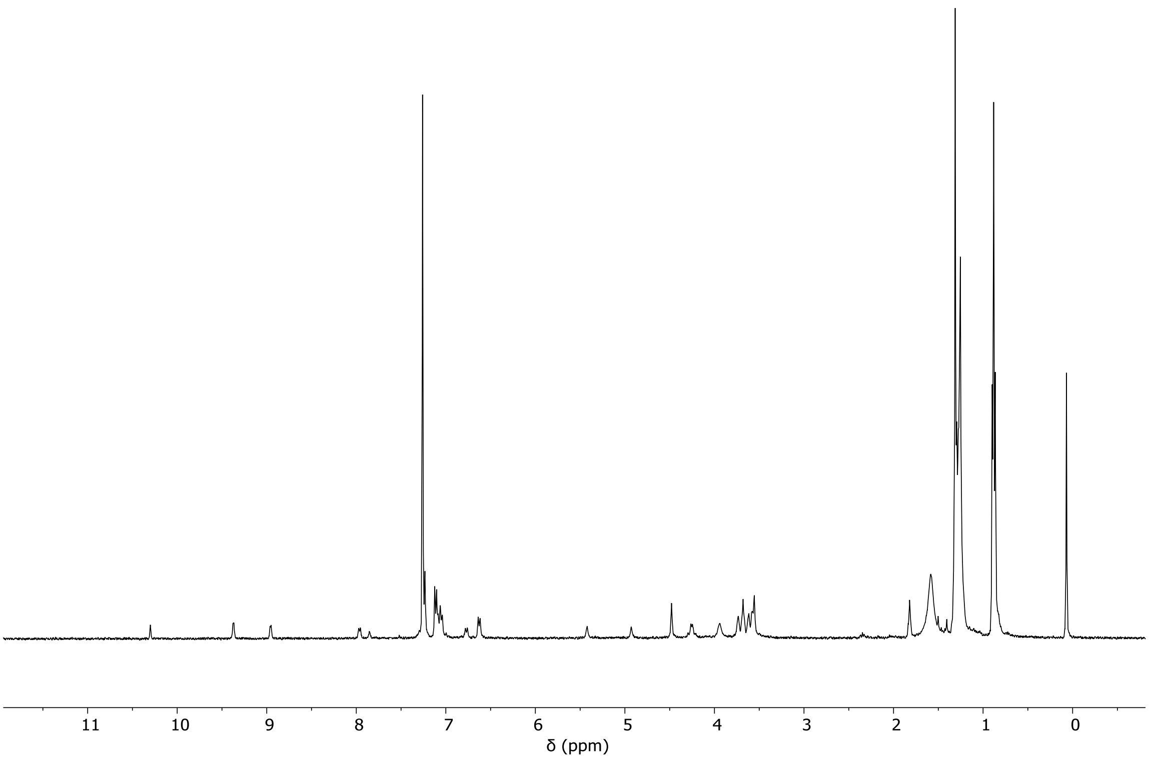

Figure S13. ^{1}H NMR spectrum (400 MHz, $CDCl_3$, 298 K) of *p*-**8•Zn**.

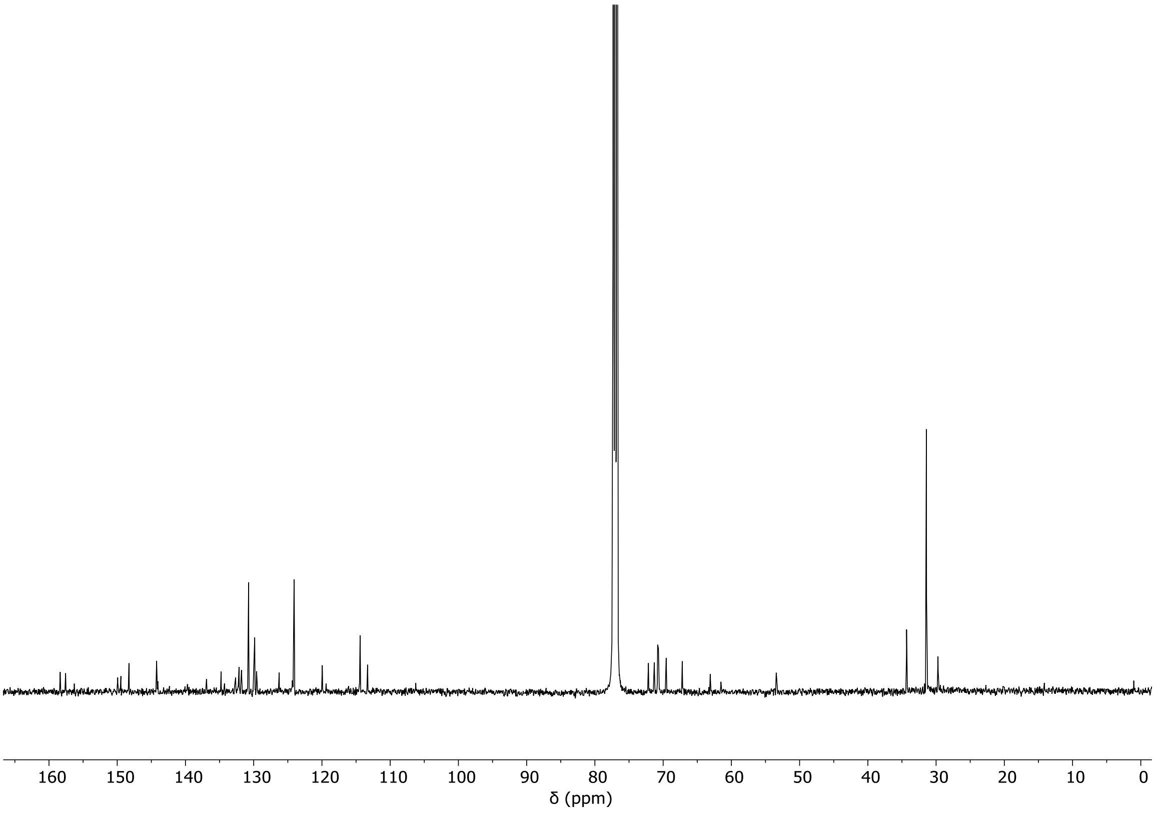

Figure S14. $^{13}C\{^{1}H\}$ NMR spectrum (126 MHz, $CDCl_3$, 298 K) of *p*-**8•Zn**.

<u>*m*-**9•Zn**</u>

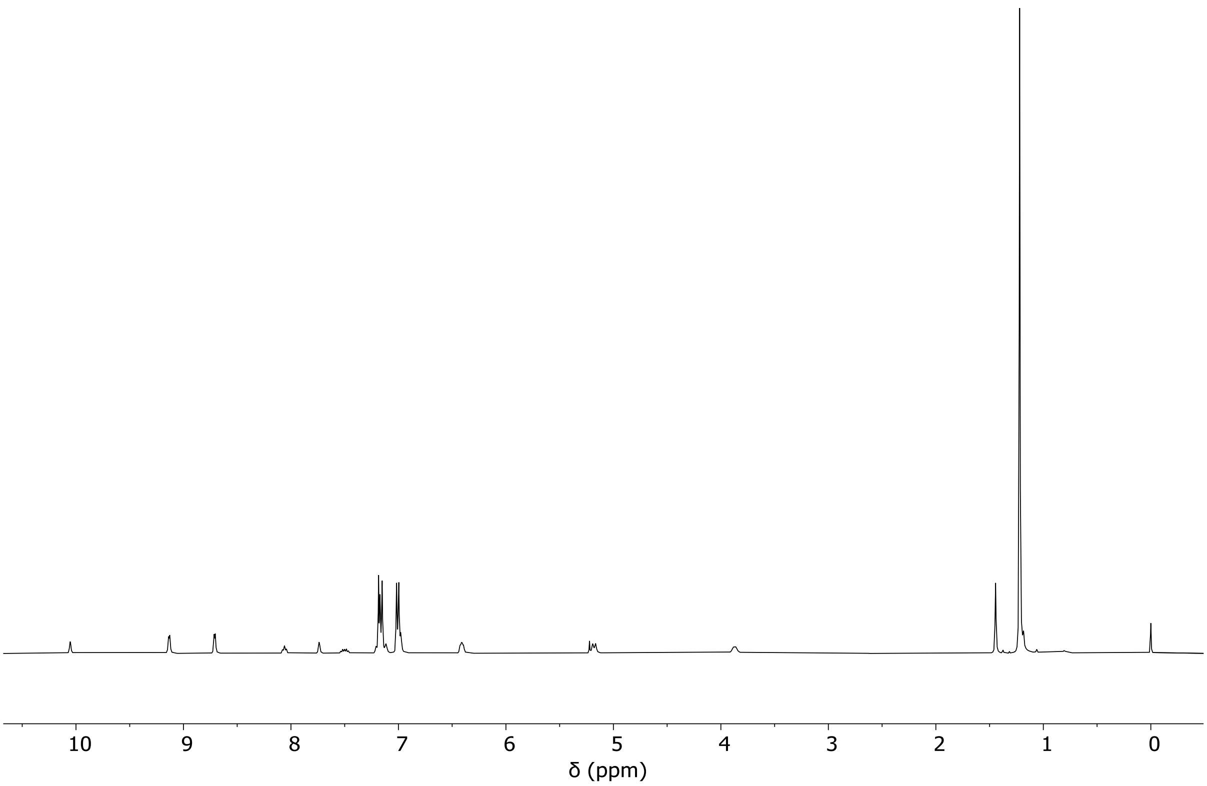

Figure S15. ^{1}H NMR spectrum (400 MHz, $CDCl_3$, 298 K) of *m*-**9•Zn**.

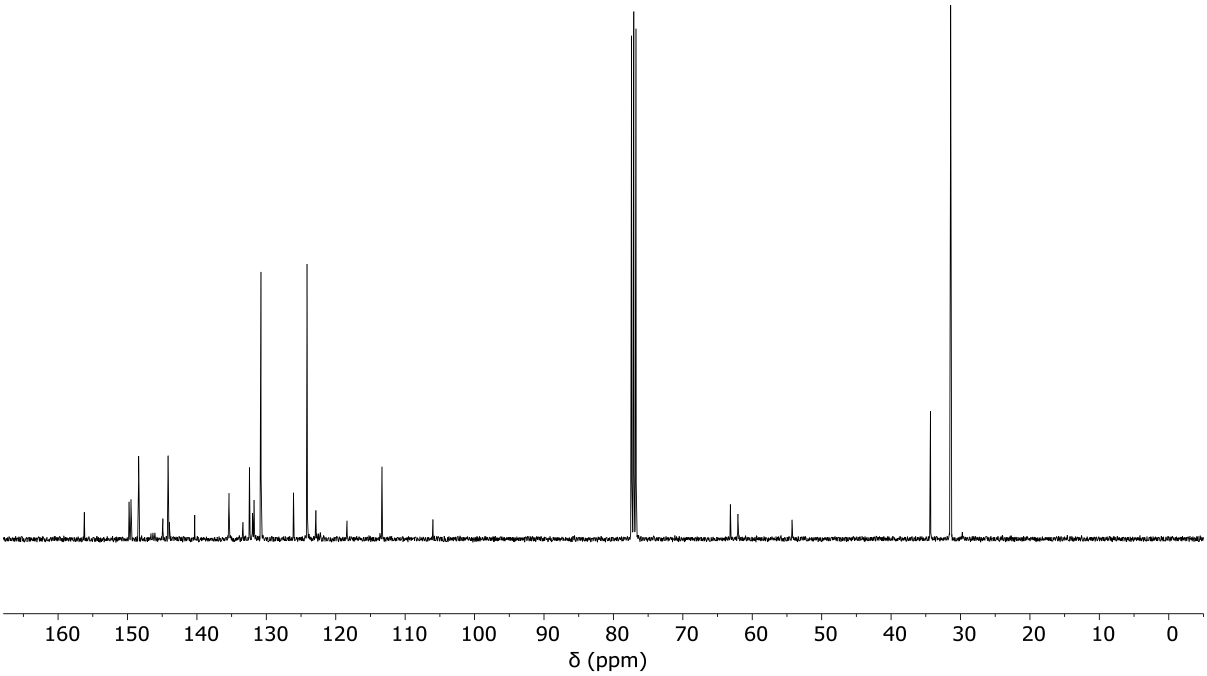

Figure S16. ^{13}C{^{1}H} NMR spectrum (126 MHz, $CDCl_3$, 298 K) of *m*-**9•Zn**.

***p*-10•Zn**

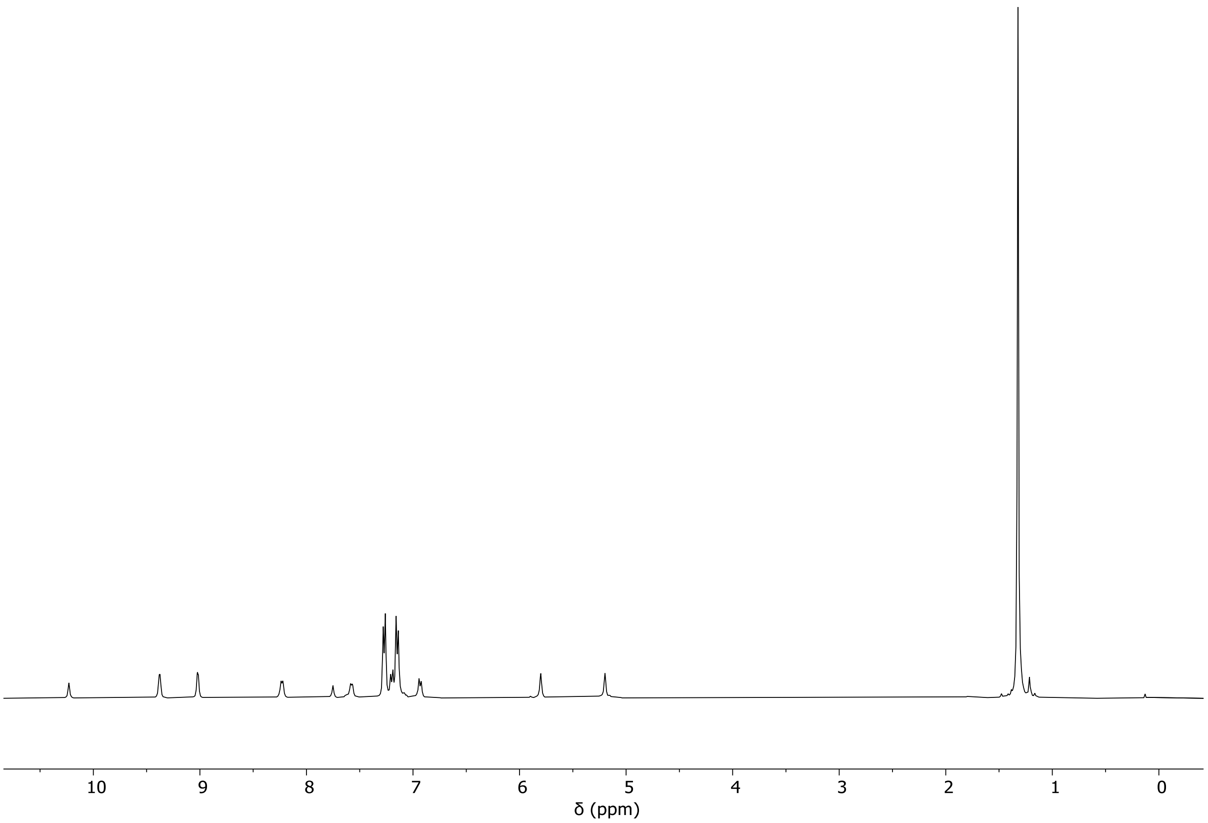

Figure S17. ^{1}H NMR spectrum (400 MHz, $CDCl_3$, 298 K) of *p*-**10•Zn**.

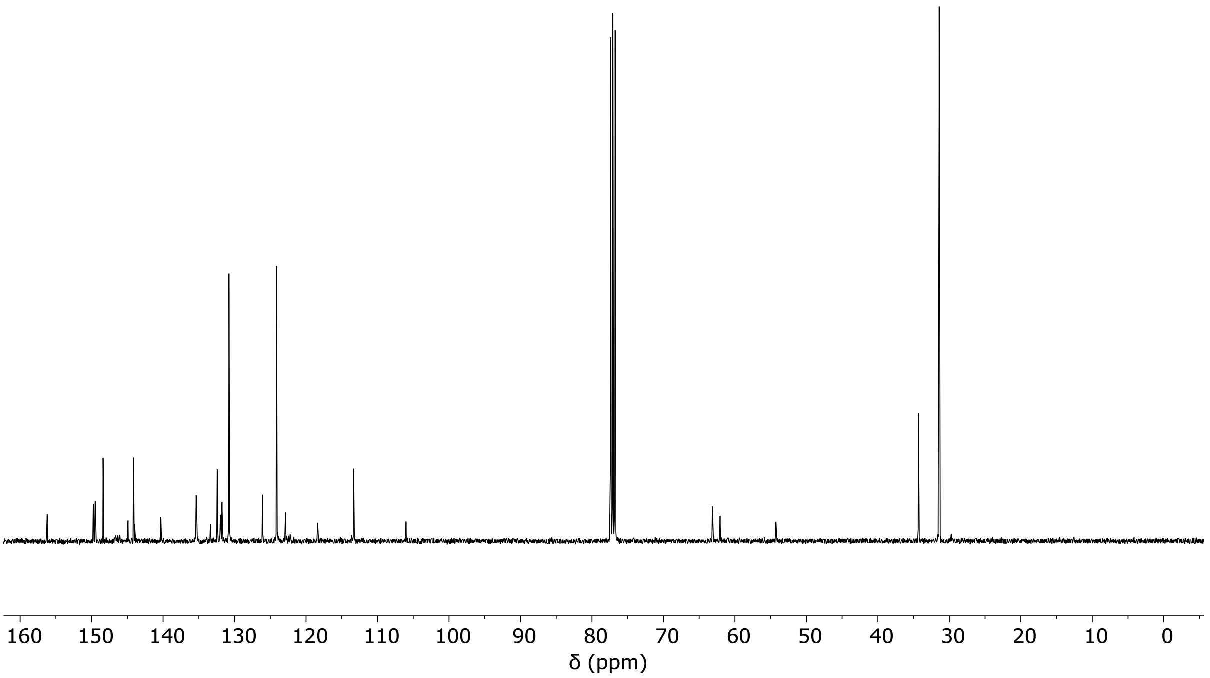

Figure S18. $^{13}C\{^{1}H\}$ NMR spectrum (126 MHz, $CDCl_3$, 298 K) of *p*-**10•Zn**.

***p*-6•H2**

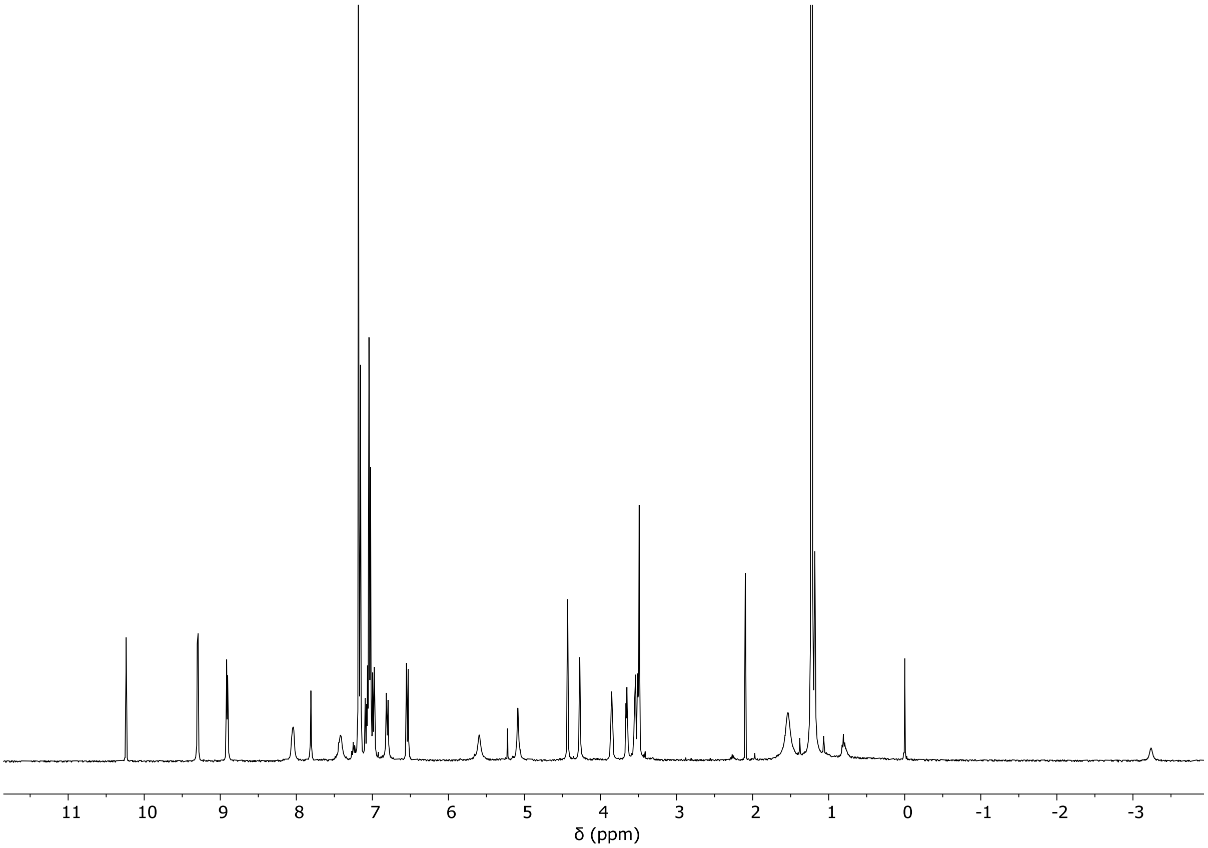

Figure S19. ^{1}H NMR spectrum (400 MHz, $CDCl_3$, 298 K) of *p*-**6•H2**.

Figure S20. ^{13}C{^{1}H} NMR spectrum (126 MHz, $CDCl_3$, 298 K) of *p*-**6•H2**.

p-**6•Ni**

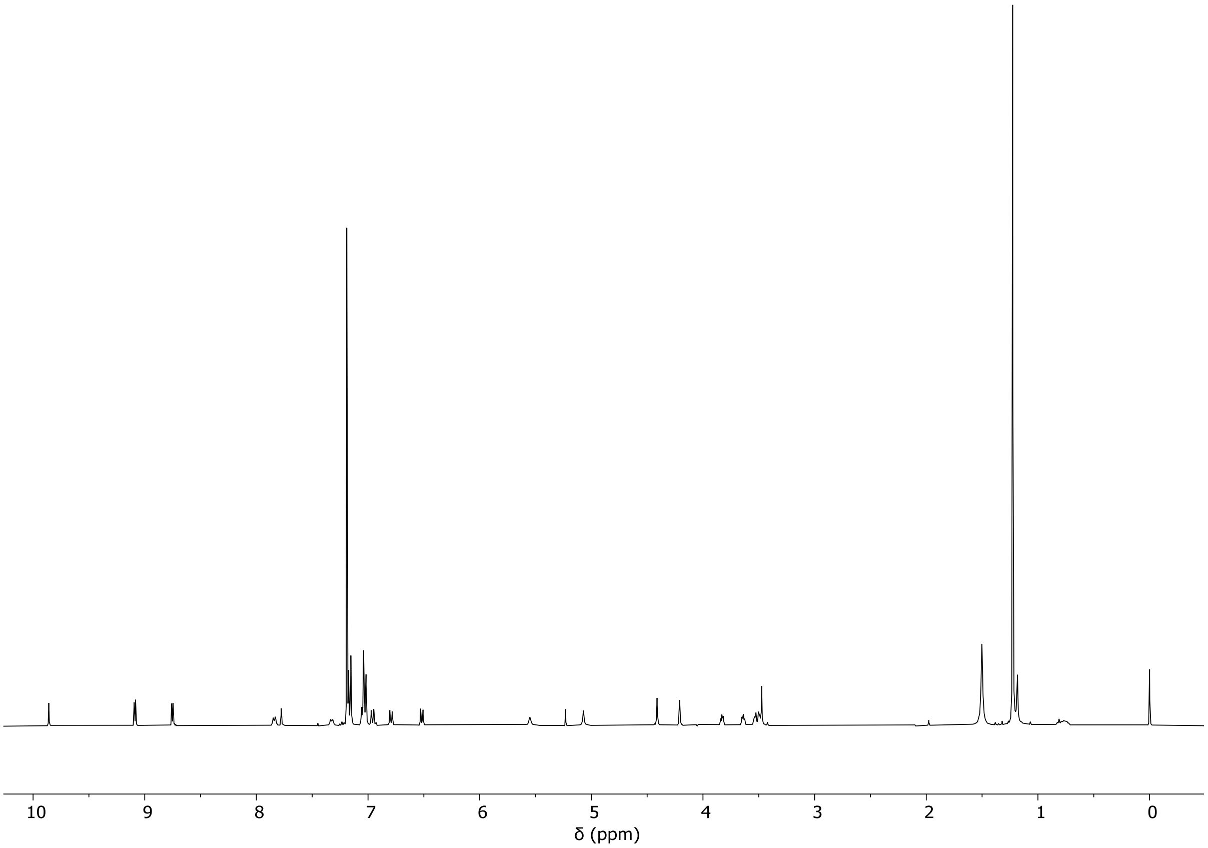

Figure S21. ^{1}H NMR spectrum (400 MHz, $CDCl_3$, 298 K) of *p*-**6•Ni**.

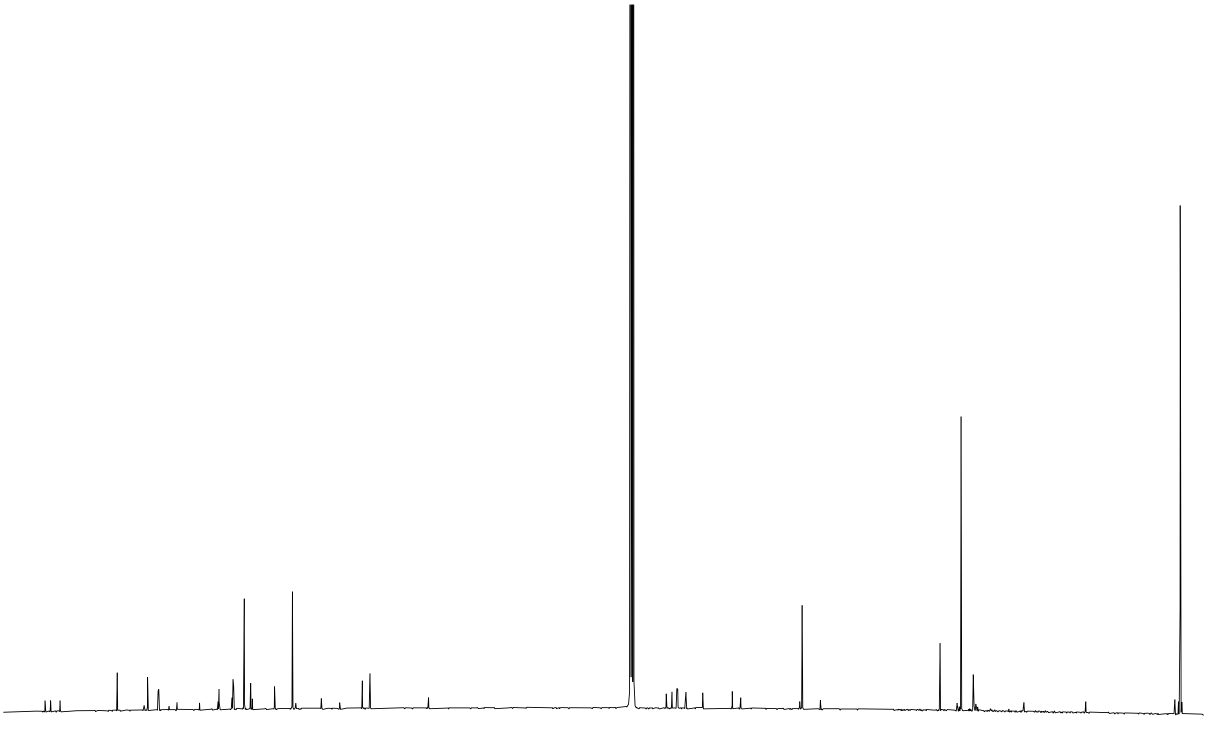

Figure S22. $^{13}C\{^{1}H\}$ NMR spectrum (126 MHz, $CDCl_3$, 298 K) of *p*-**6•Ni**.

S4 2,6-Lutidine Binding Studies

To evidence the ability of 2,6-lutidine to co-ordinate the Zn(II) metalloporphyrin core, qualitative binding studies were performed. Upon successive addition of a 250 mM 2,6-lutidine solution to a 7.5 μM solution of *p*-**6•Zn** in chloroform, a clear bathochromic shift was observed. The presence of an isosbestic point (Figure S23) is consistent with the binding of lutidine in a 1:1 binding host:guest stoichiometry. Furthermore, the ^{1}H NMR spectrum of *p*-**6•Zn** demonstrated similar perturbations to the dynamic behaviour of the [2]rotaxane upon addition of 2,6-lutidine to those observed upon pyridine addition, namely marked downfield shifts of H_4, H_8 and H_9, and resolution of the H_7 signal.

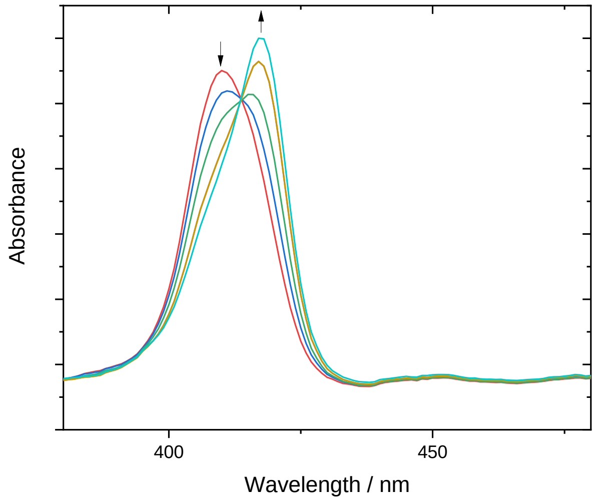

Figure S23. UV-Visible spectra of Soret band of 7.5 μM *p*-**6•Zn** solutions in $CHCl_3$, upon successive addition of 250 mM 2,6-lutidine.

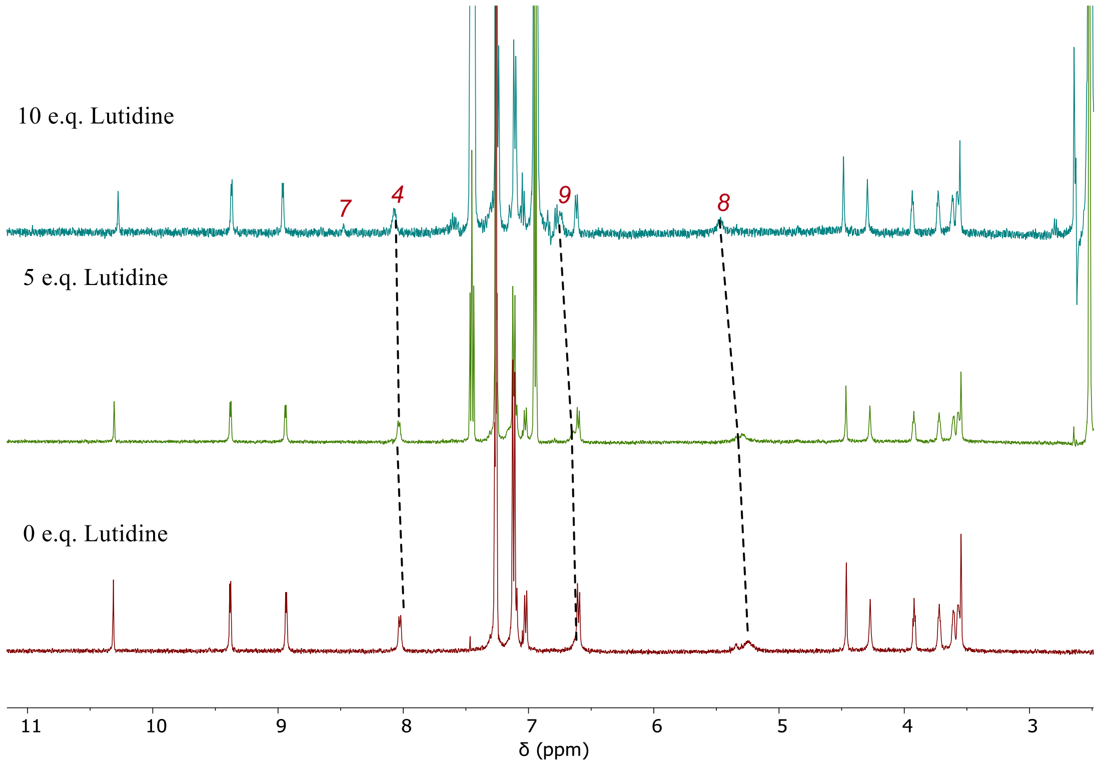

Figure S24. Stacked ^{1}H NMR spectra (500 MHz, $CDCl_3$, 298 K) of 1mM *p*-**6•Zn** upon successive addition of 50 mM 2,6-lutidine.

S5 Variable Temperature NMR Studies

Variable temperature ^1H NMR spectroscopic experiments were conducted on 1 mM $CDCl_3$ solutions of the [2]rotaxanes at 500 MHz from 233 K to 333 K. The rate constants were calculated from the spectral linewidths (in the fast-exchange regime) and peak splitting (slow-exchange regime) for porphyrin proton H_2 and thermodynamic activation parameters calculated from Eyring plots (Figure S25, Table 1).[4] A representative example of the changes in the NMR spectra , for *p*-**6•Zn**, is depicted in Figure S26.

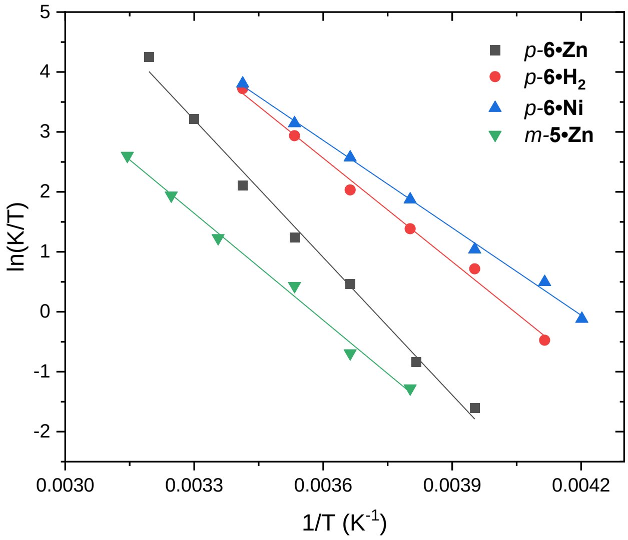

Figure S25. Eyring plots for all four rotaxane systems investigated by ^1H VT-NMR spectroscopy (1mM in $CDCl_3$).

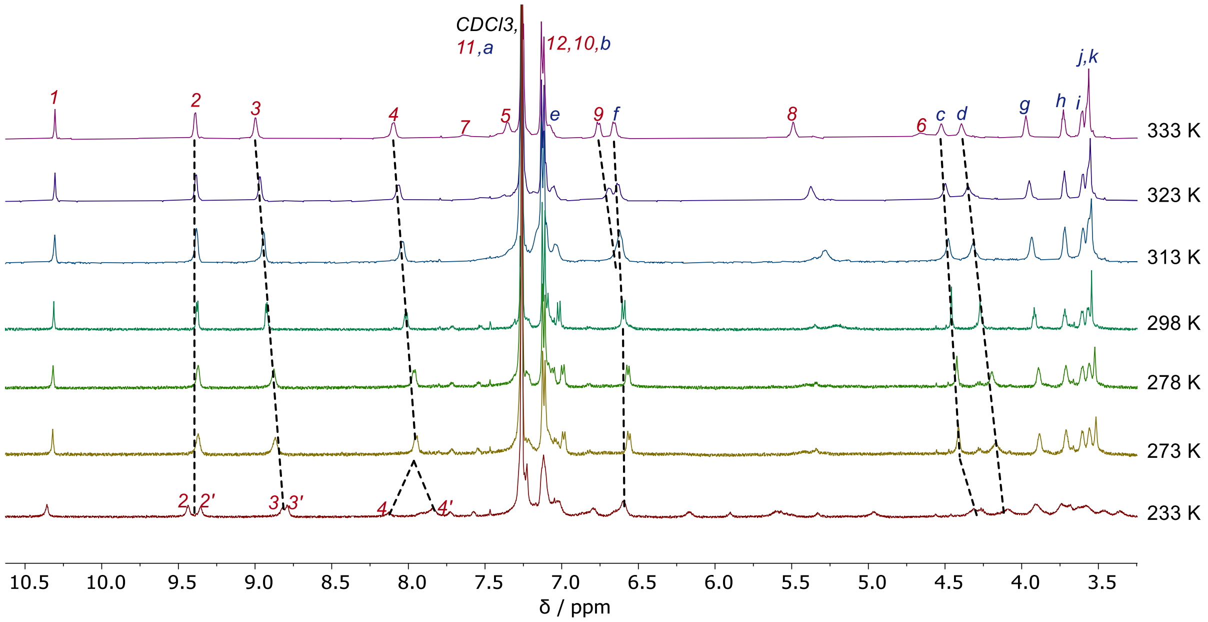

Figure S26. Stacked ^1H NMR spectra (500 MHz, $CDCl_3$) of *p*-**6•Zn** at indicated temperatures.

S6 Measurement of the 'Resting State' Bias

UV-visible titration anion binding experiments were performed at 298 K using a Horiba Duetta Fluorescence and Absorbance Spectrometer. The receptors *m*-**5•Zn,** *p*-**6•Zn.** *m*-**9•Zn** and *p*-**10•Zn**, were each dissolved in $CHCl_3$ at a concentration of 7.5 μM and their UV-visible spectra measured upon successive addition of 25 mM pyridine solution. The change in Soret band intensity for each host was fitted to a 1:1 host:guest binding stoichiometry using a global fit of all data points in the range 412-423 nm. With the assumption that the free pyridine and macrocycle pyridyl moiety bind to the Zn(II) metalloporphyrin centre in the same manner, the equilibrium constant for the resting state K_{rs} and the percentage of the rotaxane hosts in the self-included form in the absence of exogeneous pyridine were be calculated in an analogous manner to that previously reported.[5]

A representative example of the optical spectra of the Soret band upon successive addition for *p*-**6•Zn** is depicted in Figure S27.

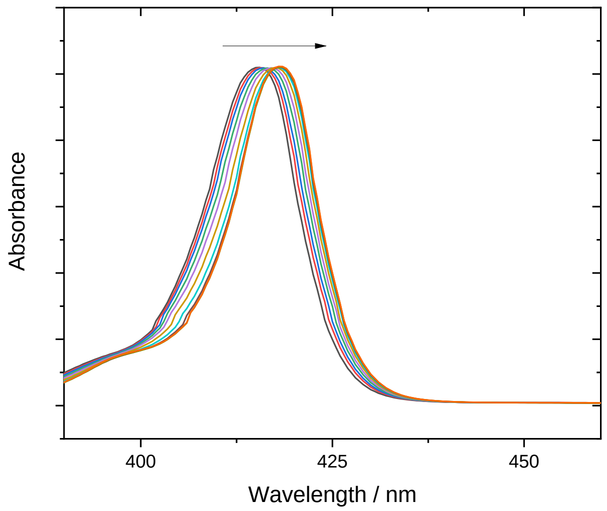

Figure S27. UV-Visible spectra of Soret band of 7.5 μM *p*-**6•Zn** solutions in $CHCl_3$, upon successive addition of 25 mM pyridine.

S7 Binding Studies of Pyridine to [3]Rotaxane

In order to further evidence that the desymmetrisation of the ^{1}H NMR spectrum of *p*-**6•Zn** upon pyridine addition arises from disruption to macrocycle translocation upon exogeneous pyridine binding, the ^{1}H NMR spectrum of *p*-**8•Zn** was recorded upon successive addition of pyridine-d_5. No desymmetrisation of the spectrum of *p*-**8•Zn** was observed (Figure S28), confirming the postulated interruption of dynamic behaviour in *p*-**6•Zn** gives rise to the loss of symmetry.

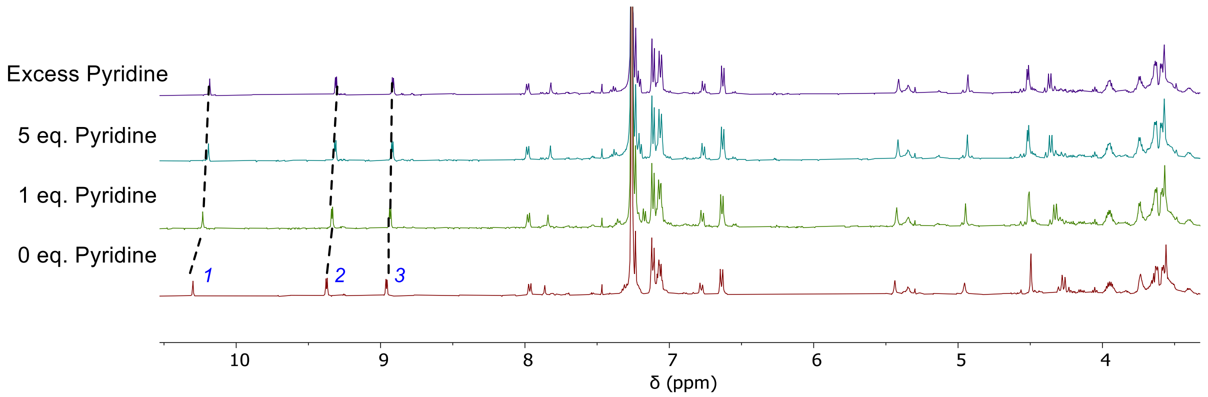

Figure S28. Stacked ^{1}H NMR spectra (500 MHz, $CDCl_3$, 298 K) of *p*-**8•Zn** upon successive addition of pyridine-d_5.

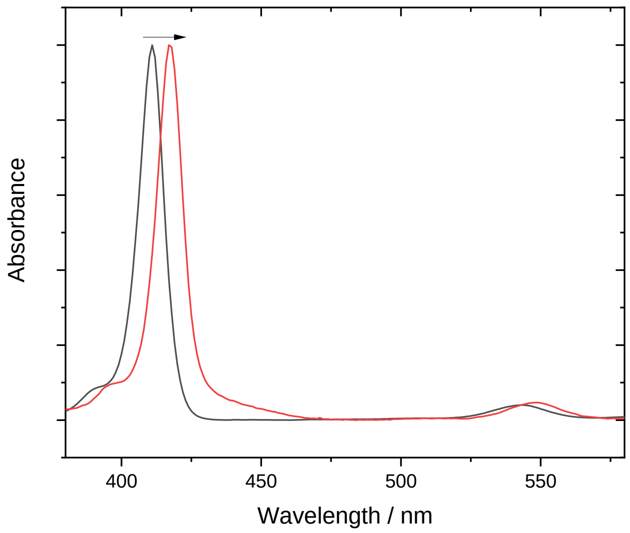

Figure S29. UV-Visible spectra of Soret and Q band of 7.5 µM *p*-**8•Zn** solutions in $CHCl_3$, before and after addition of 25 mM pyridine. Arrow indicates direction of change.

S8 Optical Titration Studies of TBAX Salt Anion Binding

UV-visible titration anion binding experiments were performed at 298 K using a Horiba Duetta. The receptor was dissolved in acetone at a concentration of 2 µM. Unless otherwise stated, the TBA salt of the anionic guest, was dissolved in the stock solution at a concentration of 100 µM. Aliquots of the guest solution were added to 1.0 mL of the host solution in a quartz glass cuvette. The sample was then mixed and the UV-visible spectra recorded upon successive additions of the TBA salt solution. The absorbance intensities at 411 nm (maximum of free host Soret band absorption), and 422 nm (maximum of host-guest complex Soret band absorption) were plotted, and the resulting isotherms globally fitted to a 1:1 stoichiometric host-guest binding model.[6]

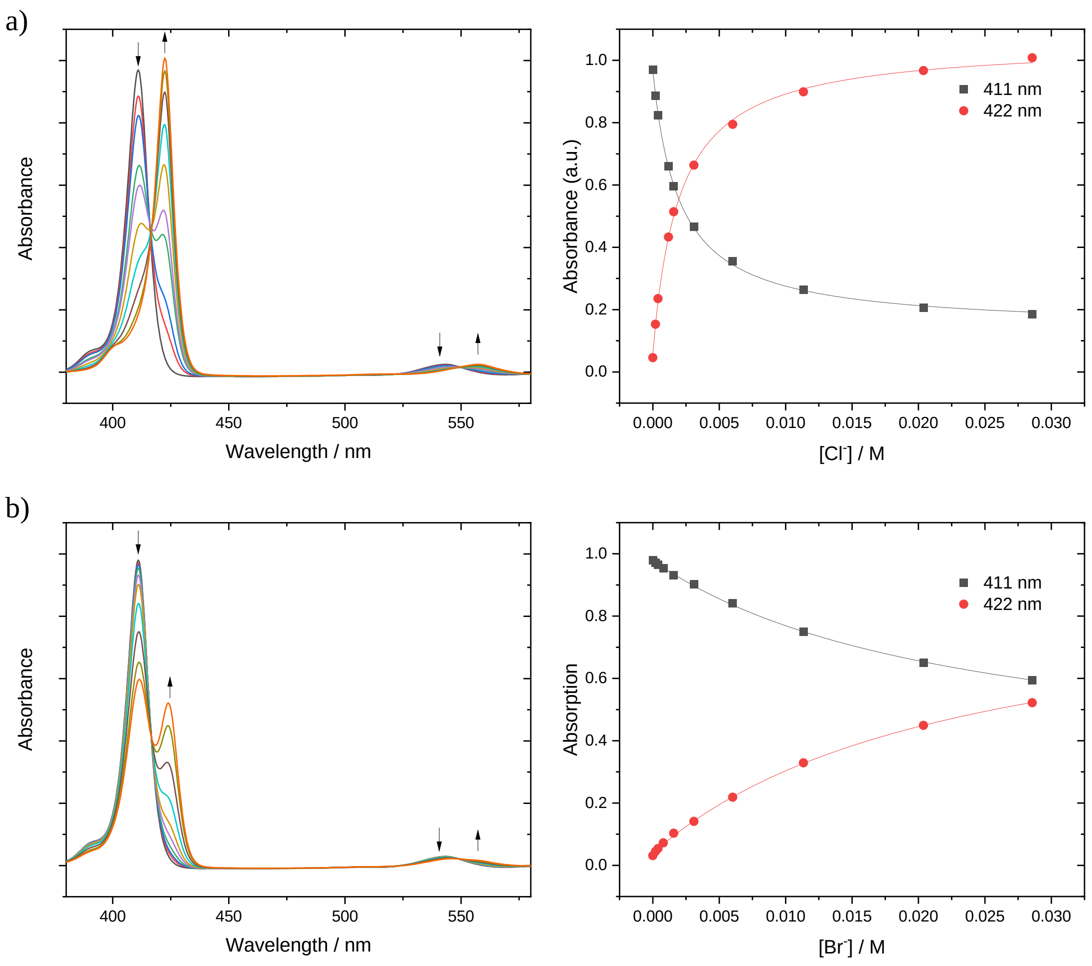

Figure S30. UV-Visible spectra of Soret and Q-bands of 2 μM *p*-**6•Zn** solutions in acetone, upon successive addition of 100 μM TBAX salts. a) X = Cl, b) X = Br.

S9 Reversibility of TBAX Salt Anion Binding

To confirm the binding of Lewis bases to the Zn(II) centre arises from non-covalent interactions, and not due to decomposition of the metalloporphyrin, silver(I) triflate (AgOTf) was added to the end solution of the titration of *p*-**6•Zn** with TBACl. Precipitation of AgCl was observed, along with a hypsochromic shift in the metalloporphyrin absorption spectrum (Figure S31), with the absorption wavelength returning to the value corresponding to *p*-**6•Zn** in the absence of any bound exogeneous Lewis base.

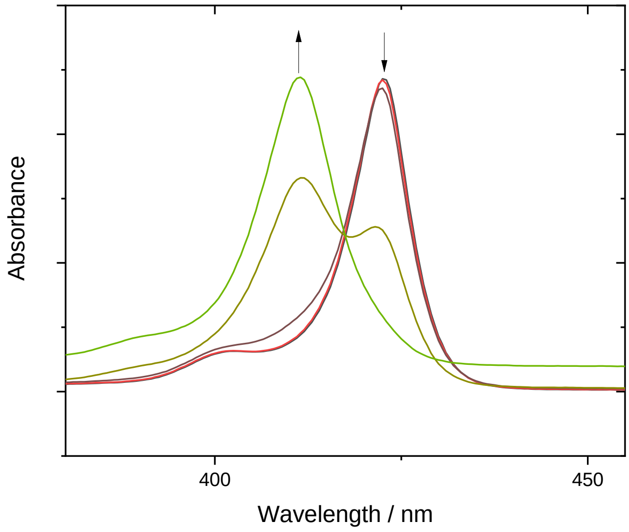

Figure S31. UV-Visible spectra of Soret band of 2 μM *p*-**6•Zn** solution with TBACl in acetone, upon successive addition of 100 μM AgOTf salt. Arrows indicate direction of change.

S10 References

[1] P. Lucio Anelli, N. Spencer, J. F. Stoddart, *Tetrahedron Lett.* **1988**, *29*, 1569-1572.

[2] D. A. Roberts, T. W. Schmidt, M. J. Crossley, S. Perrier, *Chem. Eur. J.* **2013**, *19*, 12759-12770.

[3] V. Aucagne, K. D. Hänni, D. A. Leigh, P. J. Lusby, D. B. Walker, *J. Am. Chem. Soc.* **2006**, *128*, 2186-2187.

[4] H. Eyring, *J. Chem. Phys.* **1935**, *3*, 107-115.

[5] S. W. Hewson, K. M. Mullen, *Org. Biomol. Chem.* **2018**, *16*, 8569-8578.

[6] D. Brynn Hibbert, P. Thordarson, *Chem. Commun.* **2016**, *52*, 12792-12805.
